# Proteasome-dependent protein degradation shapes developmental tempo in mouse and human neural progenitors

**DOI:** 10.1016/j.devcel.2026.07.014

**Published:** 2026-08-13

**Authors:** Shota Nakanoh, Despina Stamataki, Lorena Garcia-Perez, Chiara Azzi, Hayley L Carr, Alexandra Pokhilko, Loukik Doshi, Giulia L M Boezio, Hilary Knowles, Adriana Lamas Bancalari, Manuela Melchionda, Lu Yu, Steven Howell, Mark Skehel, David Oxley, Simon Andrews, James Briscoe, Teresa Rayon

**Affiliations:** 1 The Babraham Institute, Cambridge, United Kingdom; 2 The Francis Crick Institute, London, United Kingdom

## Abstract

The pace of embryonic development differs between mammalian species, yet the molecular basis for this remains unknown. By comparing protein dynamics in mouse and human neural progenitors (NPs), we show that protein turnover is faster in mouse NPs, driven by higher rates of protein synthesis and degradation. Human NPs exhibit longer protein half-lives, reduced proteasomal activity, and lower proteasome abundance. These differences persist in post-mitotic neurons and are also observed in the embryonic spinal cord in vivo. Pharmacological inhibition of proteasomal activity slows differentiation in mouse NPs. Conversely, enhancing proteasomal activity accelerates neuronal output in human NPs. Moreover, accelerating the degradation of the key transcriptional repressor IRX3 in mouse NPs speeds the activation of its target gene. Together, these results provide evidence that species-specific regulation of proteasome-mediated proteolysis influences the timing of neural development and suggest that evolutionary tuning of proteasomal activity contributes to differences in embryonic developmental pace.

## Introduction

The developmental mechanisms governing embryogenesis are remarkably conserved across mammals, yet developmental programmes unfold at species-specific rates^1–3^. These differences are recapitulated *in vitro* using stem cell models, enabling investigations of the mechanisms controlling developmental tempo^2–7^. Previous work implicated protein turnover, the balance of protein synthesis and degradation, as a contributor to developmental tempo^4,5^. Reduced rates of protein degradation corresponded to slower tempos in human neural progenitors (NPs) compared to mouse. Similarly, the pace of the segmentation clock during the elongation of the vertebrate axis correlates with the rate of protein turnover across mammalian species^8^.

While these findings suggest protein turnover is crucial for tempo control, the regulation of the production and degradation machineries and their dynamics during development are poorly understood. More precisely, a systematic characterisation of the similarities and differences in protein production and half-lives between species is lacking, and it is unclear whether species-specific turnover results in differences in protein abundance or is offset by compensatory changes in protein synthesis. Finally, whether manipulating the stability of proteins affects the pace of development is unclear.

Here, we address these questions by quantifying protein production and degradation in equivalent mouse and human NPs. Combining targeted protein labelling, high-resolution quantitative mass spectrometry, proteasomal activity quantifications, and protein depletion with self-labelling tags, we systematically identify species differences in protein turnover, indicating coupled differences in protein synthesis and degradation between mouse and human NPs. We find that proteasomal activity is lower in human embryonic spinal cords than in stage-matched mouse embryos and find lower abundance of proteasome complexes in stem-cell derived human NPs. Furthermore, we provide evidence that proteolytic degradation dictates developmental tempo by manipulating proteasome activity and by reducing the stability of a key regulatory protein within the gene regulatory network governing motor neuron formation in the neural tube. Together, these findings establish a causal link between protein degradation and developmental tempo, with broad implications for understanding phenotypic diversity across evolution.

## Results

### SNAP Tagged OLIG2 demonstrates differences in protein turnover between mouse and human neural progenitors

The balance between protein synthesis and degradation determines protein levels in cells. Having observed global differences in protein decay between mouse and human neural progenitors (NPs)^4^, we set out to test if similar differences were observed for specific proteins. To this end, we focused on OLIG2, an essential transcription factor for motor neuron differentiation^9,10^. To obtain data on the turnover for OLIG2 in mouse and human NPs, we tagged the C-terminus of endogenous OLIG2 in mouse and human embryonic stem cells with a HA tag and a SNAP tag, a self-labelling protein tag that enables covalent labelling by substrates^11^.

We confirmed that the differentiation dynamics of OLIG2::HA::SNAP (OLIG2-SNAP) targeted cells followed the expected pattern for OLIG2 in mouse and human NPs, with OLIG2 increasing upon Retinoic Acid (RA) and Smoothened Agonist (SAG) addition^4^. We detected OLIG2^+^ cells at day 2 in mouse, and days 4 and 6 in human, and there was a good correlation between OLIG2 antibody and HA tag (Fig. S1a). This indicated that tagging does not affect OLIG2 dynamics or function in mouse or human NPs. Next, we tested the specificity of the SNAP fluorescent substrate SNAP 647-SiR. We incubated human NPs from day 4 or day 6 of differentiation with SNAP or HALO ligands conjugated to a 647 fluorophore. We detected specific labelling with the SNAP 647-SiR ligand; the HALO-JF647 substrate did not cross-react with the hOLIG2-SNAP line (Fig. S1b).

**Figure 1:**
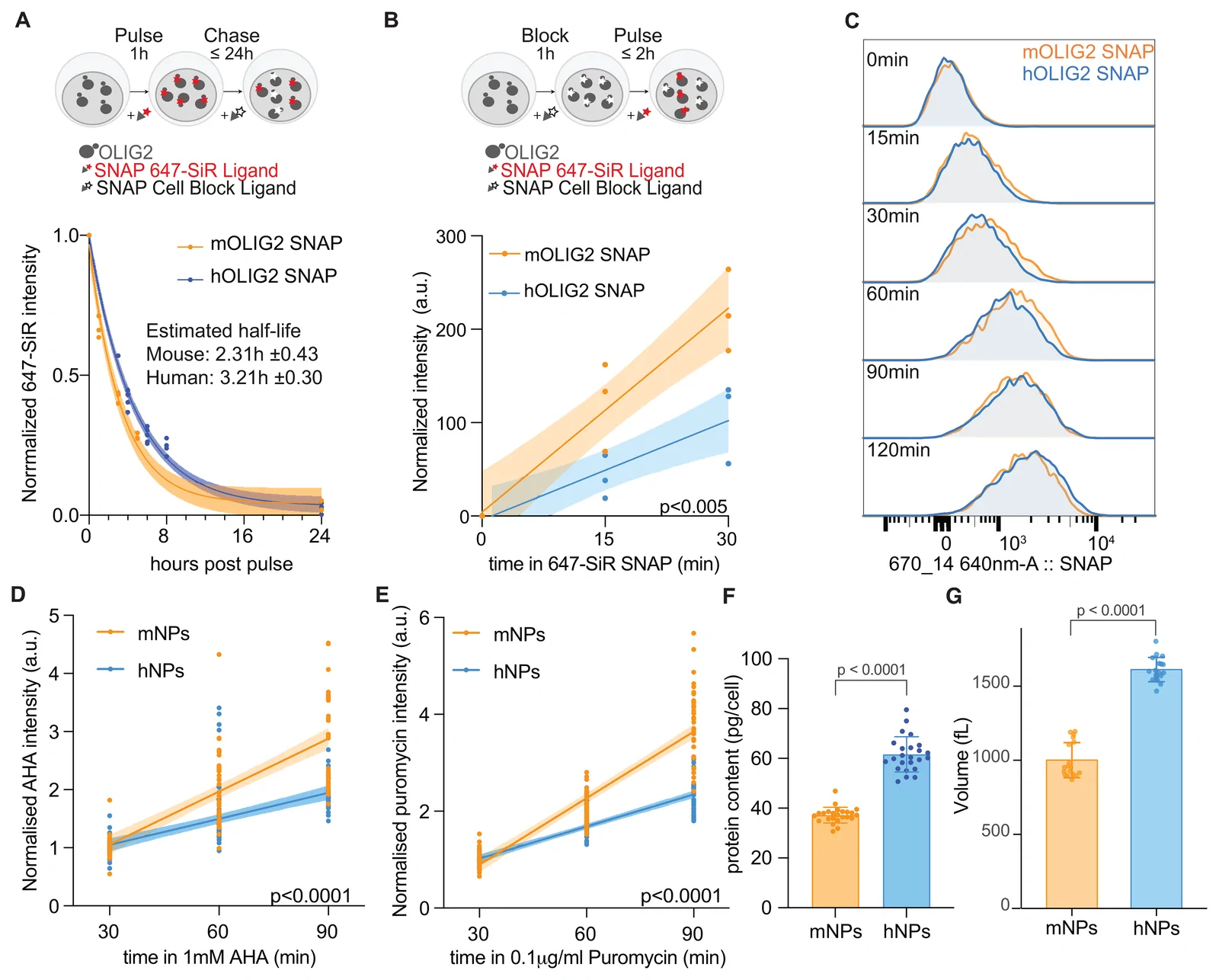
Dynamics of protein production and degradation in mouse and human neural progenitors (a) OLIG2-SNAP pulse-chase experiments in mouse (orange) and human (blue) NPs. Curves and shaded areas show exponential fit and 95% confidence intervals, respectively; (mouse n=3, human t_0 h_ n=5; t_3 h_ n=1; t_4 h_=4; t_6 h_ n=5; t_8 h_ n=4; t_24 h_ n=5). (b) Quantification of OLIG2-SNAP production in mouse and human NPs. Values show normalised intensity corrected to 0 min, with linear regression fits and 95% confidence intervals. Estimated slopes: mOLIG2 = 7.281 ± 2.26 a.u.·min^-1^; hOLIG2 = 3.544 ± 1.75 a.u.·min^-1^. P-value from ANCOVA test (n=3). (c) Representative flow cytometry analysis of 647-SiR ligand incorporation in mouse and human NPs. (d) Quantification of AHA incorporation at the indicated timepoints in mouse and human NPs, normalised to the initial value at 30 min. Dots correspond to the geometric mean intensity, with fits and 95% confidence intervals. Estimated slopes: mNP 0.030 ± 0.0023 a.u.·min^-1^; hNPs 0.015 ± 0.0016 a.u.·min^-1^. Data are shown as n = 4, with at least two technical replicates per experiment. P-value from ANCOVA test. (e) Quantification of puromycin (0.1 µg/ml) incorporation at the indicated timepoints in mouse and human NPs normalised to the initial value at 30 min. Dots correspond to the geometric mean intensity, with linear regression fits and 95% confidence intervals. Estimated slopes: mNP 0.046 ± 0.0019 a.u.·min^-1^; hNPs 0.022 ± 0.001 a.u.·min^-1^. Data are shown as n = 4, with at least two technical replicates per experiment. P-value from ANCOVA test. (f) Protein content of mouse and human NPs measured by BCA quantification. Data are shown as mean ± standard deviation. Data are shown as n = 3, including at least 20 wells. P-value from paired sample t-test. (g) Mean cell volume of mouse and human NPs estimated from coulter counter. Mean ± standard deviation. Data are shown as n = 3, including 6 technical replicates per experiment. P-value < 0.0001 from paired sample t-test.

Having established ligand specificity, we determined the half-life of OLIG2 in mouse and human NPs. First, we cultured NPs for 1h in media containing SNAP 647- SiR ligand. This was then replaced with media containing SNAP-ligands without 647 fluorophore or SNAP-cell block, a cell permeable and non-fluorescent compound that blocks the reactivity of the SNAP-tag. We observed similar trends in the decay of OLIG2 intensity in human NPs at day 4 and day 6 of differentiation (Fig. S1c), suggesting similar protein decay rates for OLIG2 during differentiation. Comparative SNAP pulse-chase analysis indicated the OLIG2 half-life was ~1.4-fold longer in human compared to mouse (mOLIG2 t1/2 = 2.31 h ± 0.43; hOLIG2 t1/2 = 3.21 h ± 0.30) (Fig. 1a). These data corroborate an increased OLIG2 stability in human compared to mouse^4^.

We next determined OLIG2 production rates for mouse and human. SNAP ligands bind to mature proteins (Fig. 1b). To distinguish between old OLIG2 and newly synthesised molecules, we cultured mouse and human NPs with SNAP-cell block to obstruct all pre-existing OLIG2 molecules present in cells. NPs were then switched into media containing SNAP 647-ligand and samples collected at intervals over the following two hours (Fig. 1b, 1c, S1d). Quantifications of the mean intensities for the ligand at various time points indicated that OLIG2 production rates in human NPs were slightly lower in comparison to mouse at 15 min and 30 min (Fig. S1d). To avoid significant loss of labelled protein by degradation during the pulse, we used timepoints that covered <10% of t1/2 for mouse and human (mOLIG2 ~15 min, hOLIG2 ~20 min), and we normalised to the initial timepoints to account for any differences in labelling efficiency between mouse and human. The accumulation of 647-SIR was ~2.05-fold faster in mNPs than in hNPs, raising the possibility of slower protein production rates in human cells (mOLIG2 = 7.281 a.u.·min^-1^ ± 2.26; hOLIG2 = 3.544 a.u.·min^-1^± 1.75) (Fig. 1b).

### Higher protein production rate in mouse versus human neural progenitors

Given the higher OLIG2 production rate in mouse, we next set out to compare global protein production rates and abundances in mouse and human NPs. We performed metabolic labelling of nascent proteins by adding the methionine analog L-azidohomoalanine (AHA)^12^ to media and determined the rate of incorporation over one hour. We treated cells with AHA and collected mouse and human NPs after 30, 60 or 90 min of treatment. The production rate was ~2.3-fold higher in mouse compared to human NPs (mNP = 0.03 a.u.·min^-1^ ±0.005; hNP = 0.014 a.u.·min^-1^ ± 0.003) (Fig. 1d). To confirm the difference in production rates between mouse and human NPs, we measured puromycin incorporation rates^13^. While both AHA and puromycin incorporate into nascent polypeptides, puromycin blocks protein synthesis and causes premature termination, with the resulting truncated polypeptides often failing to fold properly^14,15^. We treated mouse and human NPs with various concentrations of puromycin to identify the lowest concentration that allowed sufficient labelling for quantification. A puromycin concentration of 0.1 μg/ml was sufficient to detect nascent protein in mouse and human NPs without triggering a stress response, as measured by levels of EIF2A phosphorylated at Ser51^16^ (Fig. S1e-g). Side-by-side puromycin incorporation assays in mouse and human NPs confirmed a significantly higher ~2.07-fold rate of protein production in mouse (mNP= 0.045 a.u.·min^-1^ ±0.004; hNP = 0.022 a.u.·min^-1^ ± 0.002) (Fig. 1e). The puromycin results were consistent with the measurements of AHA and confirmed that the rates of protein production are higher in mouse NPs.

Higher rates of protein synthesis and degradation in mouse NPs compared to human NPs raise the question of whether total protein concentration is maintained across species or instead scales with cell size. To address this, we measured cell size and total protein content in both species. Total protein per cell was quantified using a bicinchoninic acid (BCA) assay^17^ and independently assessed using CellTrace labelling^18^ as an orthogonal approach. The BCA assay revealed a ~1.6-fold difference in protein content between mouse and human NPs (mouse 37.7 ± 8.53 pg/cell; human 61.3 ± 12.4 pg/cell) (Fig. 1f). Consistent with this, CellTrace labelling, which covalently attaches succinimidyl ester groups to protein amines, indicated higher protein levels in human NPs compared to mouse (Fig. S1h). Coulter counter measurements of cell diameter showed that hNPs are larger (Fig. S1i) and exhibit greater size dispersion compared to mNPs. On average, hNPs were 1.6-fold larger than mNPs (mNPs 1000.7 ± 118.4 fL; hNP 1611.8 ± 41.2 fL) (Fig. 1g).

Together, these results indicate that although human and mouse NPs differ in size, total protein content scales with cell size such that overall protein concentration is largely maintained, consistent with previous reports^6,19^. The higher protein synthesis and degradation rates observed in mouse NPs are compatible with a faster protein turnover regime that maintains similar protein concentrations despite differing kinetics. Importantly, while total protein content scales with size, individual protein concentrations and turnover rates are not uniformly coupled to cell size, and many proteins exhibit context- and cell-type-specific regulation^20^.

### Global proteomic differences associated with protein degradation in mouse and human NPs

Next, we asked if specific sets of proteins showed a greater species difference than others. We performed data-independent acquisition mass spectrometry (DIA-MS) of mouse and human NPs. To quantify differences in protein content per cell, we estimated protein copy numbers using the proteomic ruler approach^21^. This confirmed ~1.3-fold higher protein levels per cell in hNPs and provided approximate absolute abundances (Table S1).

**Figure 2:**
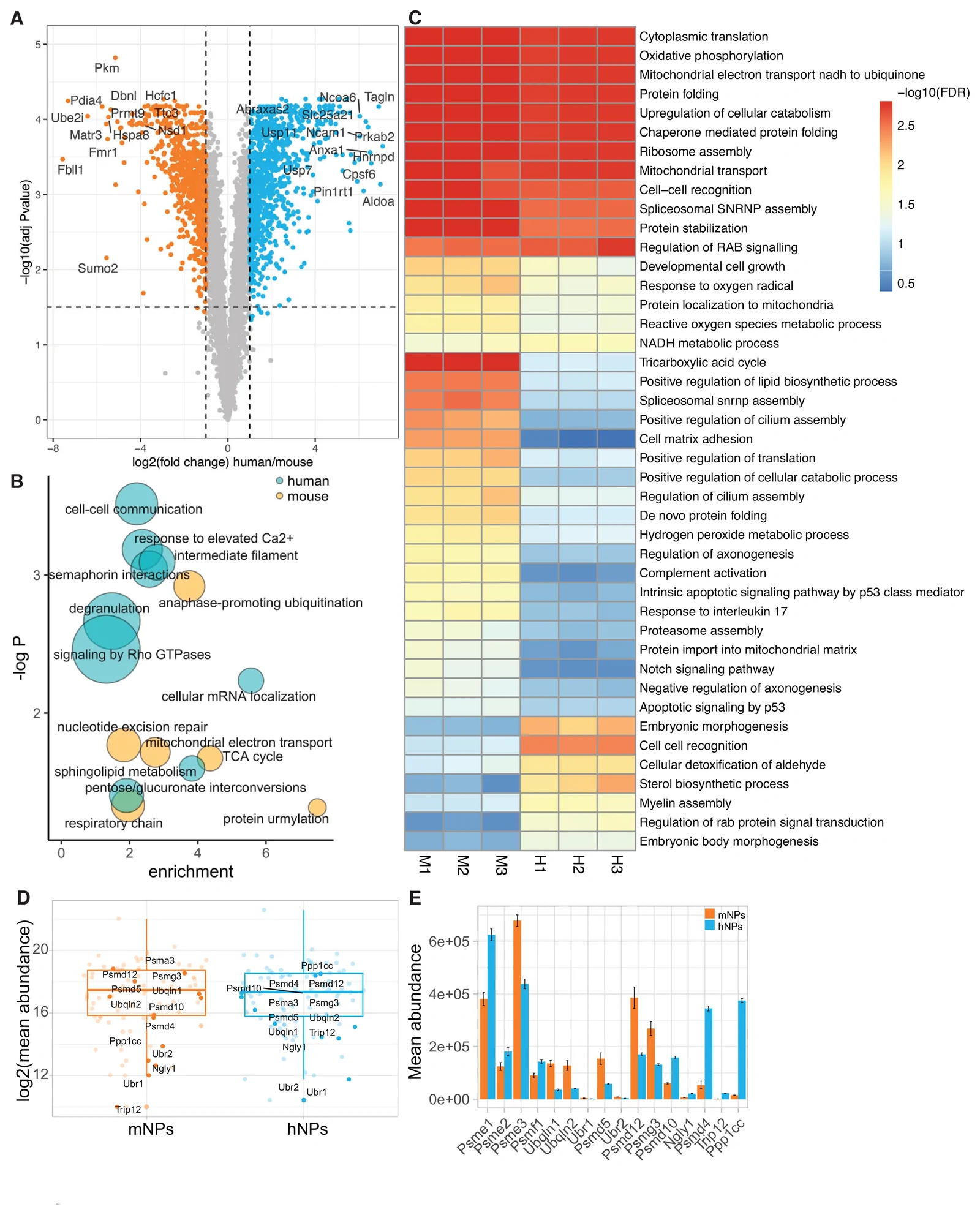
Global proteomic differences in mouse and human neural progenitors (a) Volcano plots showing 1791 Differentially Expressed Proteins (DEPs) with ≥ 2 FC and FDR ≤ 0.05 in at least 2 biological replicates. DEPs enriched in mouse (794) or human (997) depicted in orange and blue, respectively. The full list of DEPs is presented in Table S3. (b) Bubble plot depicting the top pathways identified in pathway analysis with DAVID (Table S4). The pathways enriched amongst human- or mouse-upregulated DEPs are shown in blue and orange, respectively. Bubble sizes indicate the number of DEPs in each pathway. The axes show the fold enrichment of each term compared to the background, and -log of P value from a Fisher’s test. (c) Heatmap showing single-sample gene set enrichment analysis (ssGSEA) scores for significantly enriched pathways in mouse and human NPs (Table S5). Each pathway is color coded and ordered by P-value. (d) Abundance of proteins associated to the ubiquitin-proteasome pathway in mouse and human NPs. DEPs are highlighted in darker color and labeled. (e) Mean abundance of DEPs associated with the ubiquitin-proteasome pathway in mouse and human NPs.

However, as hNPs are larger than mNPs, absolute protein copy numbers are dominated by global differences in total cellular protein content, making it difficult to resolve relative protein abundance and limiting direct cross-species comparisons. We therefore normalised protein abundances across samples to enable direct comparison between mouse and human datasets (see Methods) (Fig. S2a-e), allowing the identification of protein sets that deviate from overall proteome scaling. We identified 5718 orthologous proteins between mouse and human (Table S2), which we used for differential expression analysis. We found 1791 differentially expressed proteins with ≥ 2-fold change and FDR ≤ 0.05 (Table S3): 997 proteins upregulated in human and 794 in mouse (Fig. 2a).

Many differentially expressed proteins were related to the ubiquitin–proteasome system and ubiquitination (Fig. 2d; Table S3), and many proteasomal subunits were more abundant in mouse (Fig S2g,h). There were species-specific differences in subunits of the PA28 proteasome regulatory particles, with human NPs having increased levels of PA28αβ (PSME1/E2), and mouse NPs containing higher PA28γ/Psme3 levels (Fig. 2e). Both PA28αβ and PA28γ particles have been shown to be less efficient than the 19S canonical regulatory particle at activating the 20S proteolytic activity^22^, and could compete with one another for binding to this 20S core.

Several proteins linked to proteostasis showed increased abundance in mouse NPs, including proteasome components (Psma3, Psmd5, Psmd12), ubiquitin ligases (Smurf1, Rnf34, Cul7), ubiquitin-associated proteins (Ubqln1, Ubqln2, Ubr1, Ubr2), the proteasome assembly factor Psmg3, and the zinc finger protein Zfand1 (Fig. 2d,e, Table S2). Proteins associated with SUMOylation and protein modification pathways, including Ube2i, Sumo2, Prmt9, Nsd, and Hcfc1, were also increased in mouse NPs (Fig. 2a). Together, these observations are consistent with enhanced protein degradation capacity in mouse relative to human NPs. By contrast, the chaperones and regulatory subunits PSMD10, NGLY1, PSMD4, TRIP12, and PPP1CC were significantly upregulated in human NPs (Fig. 2d,e). The expression of deubiquitinases USP7, USP11, and ABRAXAS2, implicated in the stabilisation of proteins^23,24^, were increased in human NPs (Fig. 2a).

To explore the differences between mouse and human cells further, we performed pathway enrichment analysis^25^ (Table S4). The pathways enriched in mouse included the anaphase-promoting ubiquitination complex, a major E3 ubiquitin ligase complex with roles in the control of cell cycle and neurogenesis^26–28^, the mitochondrial electron transport chain, specifically complex III/ Ubiquinol-Cytochrome C Reductase, and enzymes of the TCA cycle (e.g. isocitrate dehydrogenase subunits IDH3G and IDH3A) (Fig. 2b). The pathways enriched in human NPs represented upregulation of cell-cell communication (e.g., NCAM1, log2FC=6; PODXL), Ca^2+^ signalling, immunity and secretion (e.g., GSN, SRP14), as well as Rho GTPase-mediated signal transduction (e.g., GNA13, GNAI1). Specific human-upregulated pathways included semaphorins, involved in axonal development (e.g., DPYSL5 hydrolase and dihydropyrimidinase CRMP1), as well as sphingolipid metabolism (e.g., SMPD4), involved in neuronal development^29,30^. The Pentose/Glucoronate interconversion pathway was upregulated in human cells. Localised in the cytoplasm, this pathway is a key route to carbohydrate synthesis. It also plays a central role in recycling NADP to NADPH, a cellular reducing agent for reductive biosynthesis and protection against oxidative stress^31^.

Differentially expressed proteins included lowly and highly abundant proteins (Fig. S2f). To complement the protein-level differential abundance analysis with a threshold-independent pathway approach, we performed single-sample gene set enrichment analysis (ssGSEA)^32^. Unlike overrepresentation analyses based on differentially abundant proteins, ssGSEA evaluates coordinated pathway enrichment within individual samples using ranked protein abundances. Significant pathway terms from either species (FDR ≤0.05) were combined, and redundant terms were collapsed using SimplifyGO^33^ based on gene overlap between pathways (Table S5). The resulting pathway heatmap showed that processes such as cytoplasmic translation, oxidative phosphorylation, and ribosome assembly were highly represented in both species. Pathways associated with the TCA cycle, proteasome assembly, developmental cell growth, and upregulation of cellular catabolism were enriched in mouse NPs, whereas pathways related to cell–cell recognition and microtubule nucleation were enriched in human NPs (Fig. 2c). Overall, the ssGSEA results complemented the differential abundance analysis and further supported enrichment of proteostasis-related and metabolic pathways in mouse NPs.

Together, the comparison of the mouse and human proteome in NPs indicated selective upregulation of proteins related to proteostasis and mitochondrial metabolism in the mouse. Proteins overrepresented in human were associated with neural phenotypes, the biosynthesis of carbohydrates, and the stabilisation of the proteome.

### Systematic differences in the stability of protein orthologs

**Figure 3:**
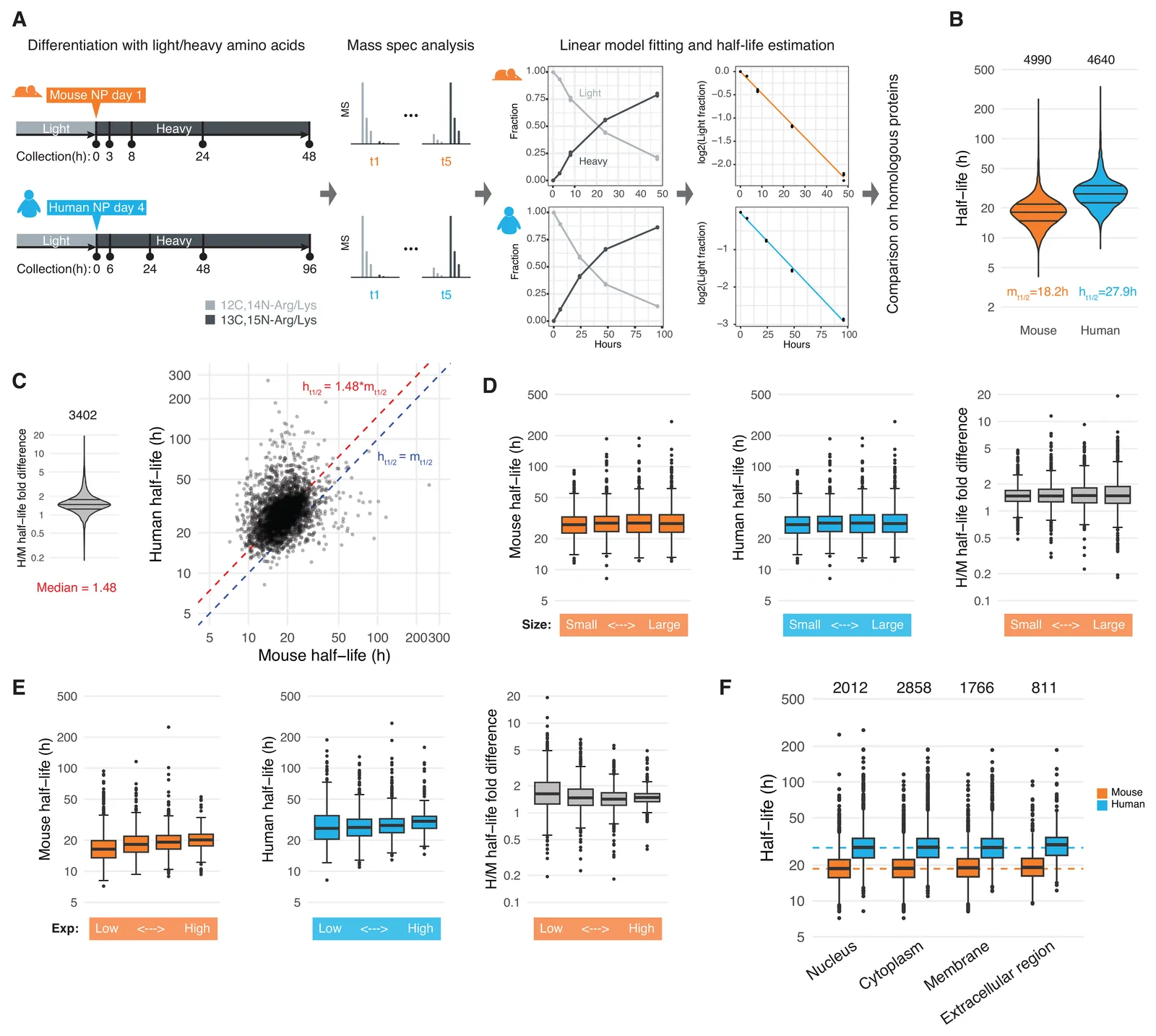
Dynamic SILAC proteomics identifies global differences in protein stability (a) Schematic of dynamic SILAC combined with quantitative proteomics. (b) Violin plot of estimated proteins half-lives in mouse and human NPs. Median values are indicated; quartiles represent the 25th, 50th and 75th percentiles. (c) Violin plot showing the fold change in protein half-lives between human and mouse NPs (left), and dot plot of 3402 homologous proteins in mouse and human (right). The blue line represents a 1:1 relationship, and the red line marks the median fold-difference. Violin plot as in (b). (d) Half-life measurements of homologous proteins divided into quartiles based on protein size (amino acid sequence length), from smallest to largest. Orange and blue box plots represent the values for mouse and human proteins, respectively. Grey box plots indicate the fold difference in half-lives in human versus mouse. Box-plot display median (centre line), interquartile range (box), whiskers (1.5× IQR), and outliers (points). (e) Quartiles of measured half-lives homologous proteins partitioned according to protein abundance (average total expression level), from low to high. Orange and blue box plots correspond to mouse and human values selected for partitioning, respectively. Grey box plots indicate the fold difference in half-lives in human versus mouse. Box-plot as (d). (f) Half-lives of homologous proteins associated with four GO terms in cellular component. Dashed lines indicate median half-lives of mouse and human homologues. Box-plot as in (d).

Having established a genome-wide difference in the proteome between mouse and human NPs^4^, we proceeded to profile protein turnover in mouse and human to determine the identity of specific proteins and pathways that exhibit similar or divergent protein dynamics between species. We performed dynamic SILAC (Stable Isotope Labelling by Amino acids in Cell culture) coupled to mass spectrometry in mouse and human NPs to estimate half-lives of individual proteins at a proteome-wide scale^34^. The labelling was centered on equivalent timepoints in mouse and human NPs based on our previous stability analysis^4^, and the collection of five timepoints per species was distributed according to the expected stability differences between mouse and human. The media was switched to heavy lysine and arginine isotopes 24 hours before day 2 in mouse. For human, we started labelling on day 4, 48 hours before human NPs reached the stage assayed (Fig. 3a). The samples were analysed by LC-MS/MS at each timepoint, and the degradation rate constants for individual proteins were calculated (see Methods). We determined the half-lives of 4990 and 4640 proteins in mouse and human, respectively (Fig. 3b, Table S7, S8). Their median half-lives were 18.2 h and 27.9 h, respectively. To obtain more detailed insight into protein features associated with differences in stability in the mouse and human datasets, we performed gene set enrichment analysis (GSEA) across all GO terms (Fig. S3a,b). Structural proteins were enriched for long half-lives in both mouse and human, as expected. Furthermore, proteins with metabolism- and translation-related functions, and proteins located in the mitochondria and ribosomes were more stable on average, especially in mouse (Table S9, S10).

We further compared the half-lives of 3402 homologous proteins detected in both mouse and human datasets (Fig.3c, Table S11). Half-lives of human homologues were generally longer than those of mouse homologues, showing a median fold difference of 1.48 (Fig. 3c), consistent with the measurement for OLIG2 (Fig. 1a). To investigate whether specific features of proteins determine their stabilities, we first split the 3402 homologous proteins into quartiles based on their size (amino acid sequence length) or abundance (average total expression level). Each quartile exhibited similar species-specific trends of half-lives and interspecies fold differences despite their sizes (Fig. 3d; Fig. S3c,d). We observed a moderate correlation between protein abundance and half-lives, with more abundant proteins tending to be more stable; however, the half-life fold differences between species were similar irrespective of expression levels (Fig. 3e; Fig. S3e,f). These results indicated that the overall differences in protein stability between mouse and human NPs are independent of protein size and abundance.

To examine if differences in the subcellular localisation of proteins affected half-life, we analysed the dataset categorised by cellular compartments (see Methods). Proteins assigned to the nucleus, cytoplasm, membrane or extracellular compartment showed similar median half-lives within each species (Fig. 3f). Consequently, the observed stabilisation of the human proteome compared to the mouse occurs in all cellular compartments. We then categorised proteins based on their molecular functions by referring to GO terms (Fig. S3g). Although most of the groups followed the overall trend, proteins classified as “Structural molecule activity”, such as TUBB, RPL32 and LMNA, and “ATP-dependent activity” appeared longer-lived, while the groups “Transcription regulator activity”, including transcription factors like POU3F2, YAP1 and ZEB2, and “Molecular adaptor activity” were short-lived in comparison with the overall median (Table S12). Nevertheless, the fold difference in half-life between the species was preserved for the proteins. Together, the results indicate that proteins in diverse biophysical properties, functional categories, and subcellular locations have similar stability differences between mouse and human NPs.

Given that our earlier comparative proteomics analysis had indicated several differences in the proteasome between the two species, we reasoned that the observed differences in proteome half-lives could result from differences in the stability of specific proteasome components, which together accounts for ~70% of cellular protein degradation^35^. We grouped proteins associated with the proteasome into three categories: core proteasomal components, proteasomal regulatory components, or proteasomal activators/inhibitors. The classification was based on a curated list generated by the proteostasis consortium^36^. We then compared the median half-lives of these protein categories with the median proteome half-life in mouse and human. Core particles followed the median trend for mouse and human (Fig. S3h). By contrast, regulatory particles were more stable than average in both species, especially in mice (Fig. S3h). For proteins in the “activator/inhibitor” category, human proteins were less stable relative to the human median, but this pattern was not observed in mouse (Fig. S3h,i). For example, the half-life of human PSME3 was shorter than the human median, whereas mouse Psme3 aligned with the mouse median (Fig. S3i). The increased degradation rate in human PSME3 explains the significant enrichment of Psme3 in mouse in the global dataset (Fig. 2e). Similarly, the proteasome regulator Psmf1^37^ was less stable than the mouse median, but human PSMF1 showed stability comparable to the human median. Global proteomic quantification indicated that Psmf1 was expressed at low levels overall, but at higher levels in human than in mouse (Fig. 2e), consistent with the measured dynamics.

Together, these results identify species-specific differences in the stability of proteasome activators and inhibitors. They suggest that the differential availability of these regulators could contribute to the differences in protein stability between mouse and human.

### A role for proteasome-dependent protein degradation in mouse and human neural cells

**Figure 4:**
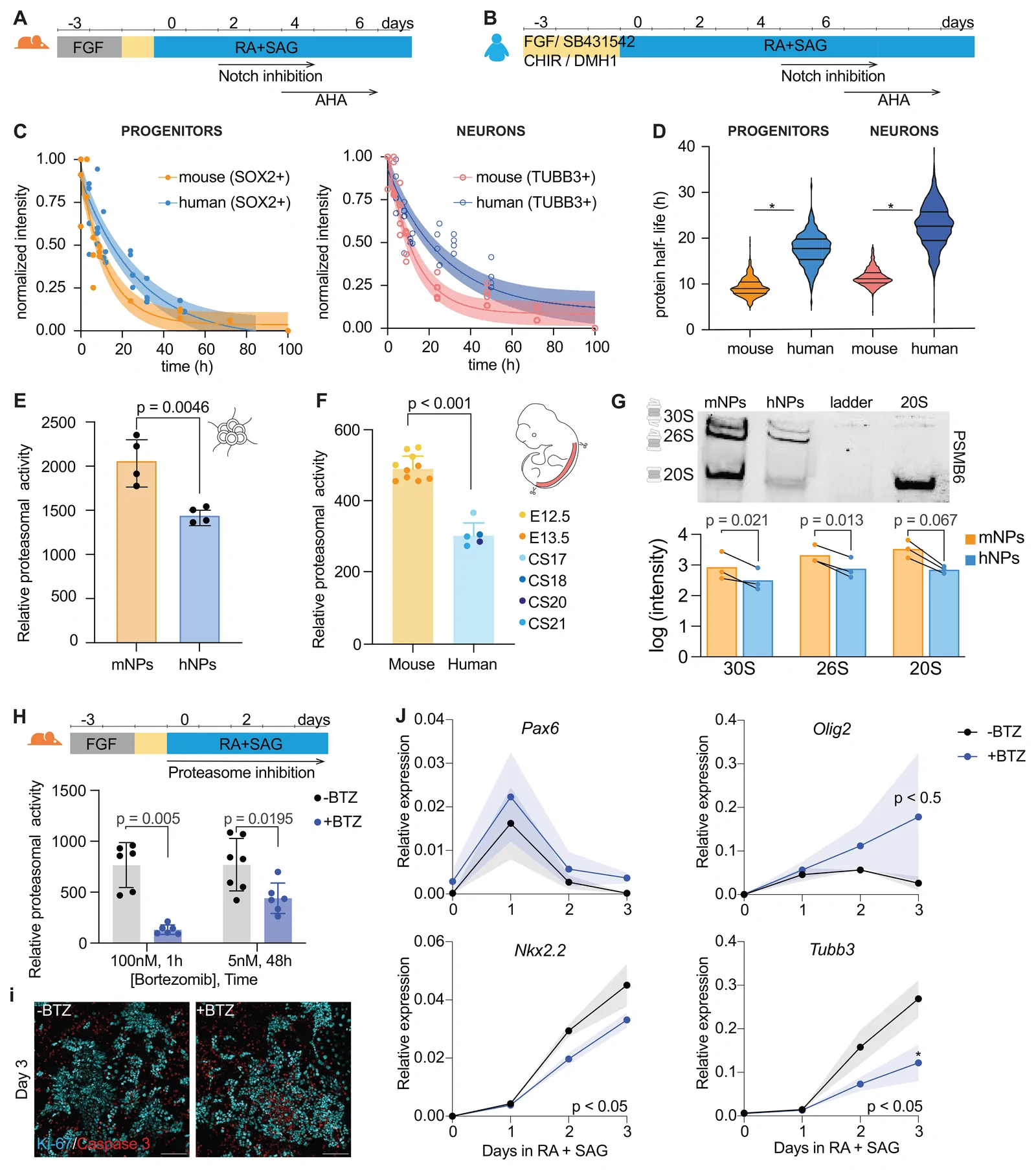
Proteasomal activity is higher in mouse neural progenitors (a, b) Schema of the experimental design for neuronal differentiation from ESCs. A Notch inhibitor (10mM DBZ, gamma secretase inhibitor) was added to increase NPs differentiation into post-mitotic neurons. In mouse, treatment was administered from day 2 - 4, followed by AHA pulse -chase experiments on day 4 to measure protein stability in progenitors and neurons (a). In human NPs, cells were treated for 48 h with the Notch inhibitor starting at day 4, and protein stability measured after treatment (b). (c) Normalised AHA intensity in mouse and human NPs (mouse, orange; human, blue) and post-mitotic neurons (mouse, red; human, dark blue) across timepoints. Curves and shadowed areas show best exponential fit with 95% confidence intervals; (mouse n = 2, human n = 2 in duplicates). (d) Half-live estimation of the proteome in mouse and human NPs (mouse, orange; human, blue) and post-mitotic neurons (mouse, red; human, dark blue) for datasets in (c). Violin plots show the 25th, 50th (median) and 75th percentiles. Statistical significance (*) indicates < 5% of overlap between distributions of parameter estimates (mouse n = 2, human n = 2 in duplicates). (e) Fluorescence intensity of proteasome chymotrypsin-like activity of using a fluorescent peptide substrate. Lines show mean values at the measured timepoints, and shaded areas represent the standard deviation (n=4). (f) Proteasomal activity measured as the slope of fluorescence increase in (e) in mouse and human NPs. Data are mean ± standard deviation; p-value from unpaired t-test (n=4). (g) Representative native gel analysis of mouse and human NPs to detect proteasome configuration. Membranes were immunoblotted for PSMB6, and purified 20S proteasome served as control. Proteasome forms (30S, 26S, 20S) are depicted on the left. Log-intensity quantifications are shown below. Data are mean ± standard deviation; lines represent biological replicates; p-value is from paired t-test (n=3). (h) Schema of the experimental design for Bortezomib (BTZ) treatment on mouse neural differentiations from ESCs, and proteasomal activity measurements at the indicated timepoints and concentrations. Treatment was administered from day 0 - 3. Data are mean ± standard deviation; p-value from paired t-test (n=3 in duplicates). (i) Confocal images of Day 3 neural progenitors treated with or without Bortezomib (BTZ) and stained for Ki-67 (cyan) and cleaved caspase-3 (red). Scale bar, 100 μm. (j) RT-qPCR of *Pax6, Olig2, Nkx2.2* and *Tubb3* in mouse neural differentiations ± Bortezomib (BTZ). Data represent mean ± standard error of the mean; two-way ANOVA followed by with Sidak’s multiple comparisons test. Reported P-values from ANOVA results, asterisks indicate statistical significance from the post hoc test (n = 4).

The faster cell cycle in mouse compared to human^4^ raised the possibility that the lower rate of protein turnover in human NPs could arise from lower cell division rates resulting in a lower rate of passive dilution. To test this, we set out to compare protein stability in non-dividing cells by inducing the generation of post-mitotic neurons *in vitro* from mouse and human NPs (Fig. 4a,b). Treatment with the gamma-secretase inhibitor Dibenzazepine (DBZ), which inhibits the activation of Notch pathway, increased the production of TUBB3^+^ neurons at the expense of SOX2 expressing NPs^38^ (Fig. 4a,b, Fig. S4a,b). Measurements of protein stability in these conditions by AHA pulse-chase labelling^4^ showed that the half-life in mouse post-mitotic neurons was ~10 h (t1/2 = 11.45 ± 1.8 h), and ~20 h in human neurons (t1/2 = 22.93 ± 4.58 h) (Fig. 4c,d, Fig. S4c). These rates were similar to those measured in NPs (mouse NPs t1/2 = 9.3 ± 1.99 h; human NPs t1/2 = 17.57 ± 3.6 h). These results indicate that protein degradation is slower in human neurons than mouse neurons and support the conclusion that active protein degradation mechanisms drive differences in protein stability between mouse and human.

A role for active protein degradation underlying species differences in protein turnover predicts higher proteasome activity in mouse NPs than in human NPs. This is supported by the global and SILAC proteomic analyses that indicated differences associated with protein degradation (Fig. 2b, Fig. S3h,i). To test this directly, we used a fluorogenic assay to measure chymotrypsin-like proteasomal activity^39,40^ in mouse and human NPs at equivalent developmental stages, normalised to total protein amount. We observed 1.43-fold higher activity in the mouse compared to human (Fig. 4e, Fig. S4d). We also observed higher trypsin-like and caspase-like proteasomal activities, the other two major catalytic activities of the proteasome, in mouse NPs (Fig. S4e), a difference reproduced in two different mouse and human embryonic stem cell lines (Fig. S4f). Moreover, developing mouse spinal cords exhibited higher proteasomal activity than stage-matched human samples *in vivo* (Fig. 4f, S4g).

Higher proteasomal activity in mouse could reflect higher abundance of active proteasomes overall or altered proteasomal configuration. Using native gel western blotting^41^ we observed increased 30S, 26S, and 20S proteasomes complexes in mNPs under equal total protein loading conditions (Fig. 4g). In both species, the 26S and 20S proteasomes dominated, with a relative enrichment of 20S in mNPs. This agrees with proteomic data showing higher proteasomal subunits levels in mNPs (Fig S2g,h), indicating that increased proteasome abundance contributes at least in part to the elevated protein degradation in mouse.

To test if decreasing proteasomal activity leads to a delayed differentiation in mouse, we treated mouse differentiations with low doses of the proteasome inhibitor Bortezomib (BTZ)^42^ from the time of RA and SAG addition. Cells treated for 1 h with 100nM of BTZ showed a significant decrease of proteasomal activity but died within 24 h. Cells treated with 5nM BTZ survived for four days and showed decreased proteasomal activity, albeit less marked than for 100nM BTZ (Fig. 4h). Cellular fitness under 5nM BTZ remained largely comparable to untreated neural progenitors, as assessed by similar proportions of actively proliferating Ki-67^+^ NPs (Fig. 4i, Fig. S4l) and only a modest increase in apoptosis after 3 days of treatment (still comparable to untreated controls) (Fig. 4i, Fig. S4m), supporting the use of this condition to assess differentiation effects. Nevertheless, there was a decrease in the percentage of NPs (SOX2^+^) and neurons (TUBB3^+^) 3 - 4 days after treatment suggesting deleterious effects of prolonged treatment (Fig. S4h,i). This is possibly due to proteotoxic stress and limited our analysis on the pace of differentiation. We therefore restricted our analysis to 3 days of differentiation in RA and SAG. The fraction of early neural progenitor markers PAX6^+^ and OLIG2^+^ cells was unchanged upon 5nM BTZ treatment (Fig. S4j,k), but RNA levels and its protein intensity were higher for the two markers up to 72 h after treatment in the few surviving cells (Fig. 4j, S4n). By contrast, the expression of the late differentiation markers Tubb3 and Nkx2.2 were decreased (Fig. 4j). This indicates a moderate stabilisation of early transcription factors within the gene regulatory network in response to reduced protein degradation via proteasomal inhibition, accompanied by a slowdown of mouse motor neuron differentiation.

**Figure 5:**
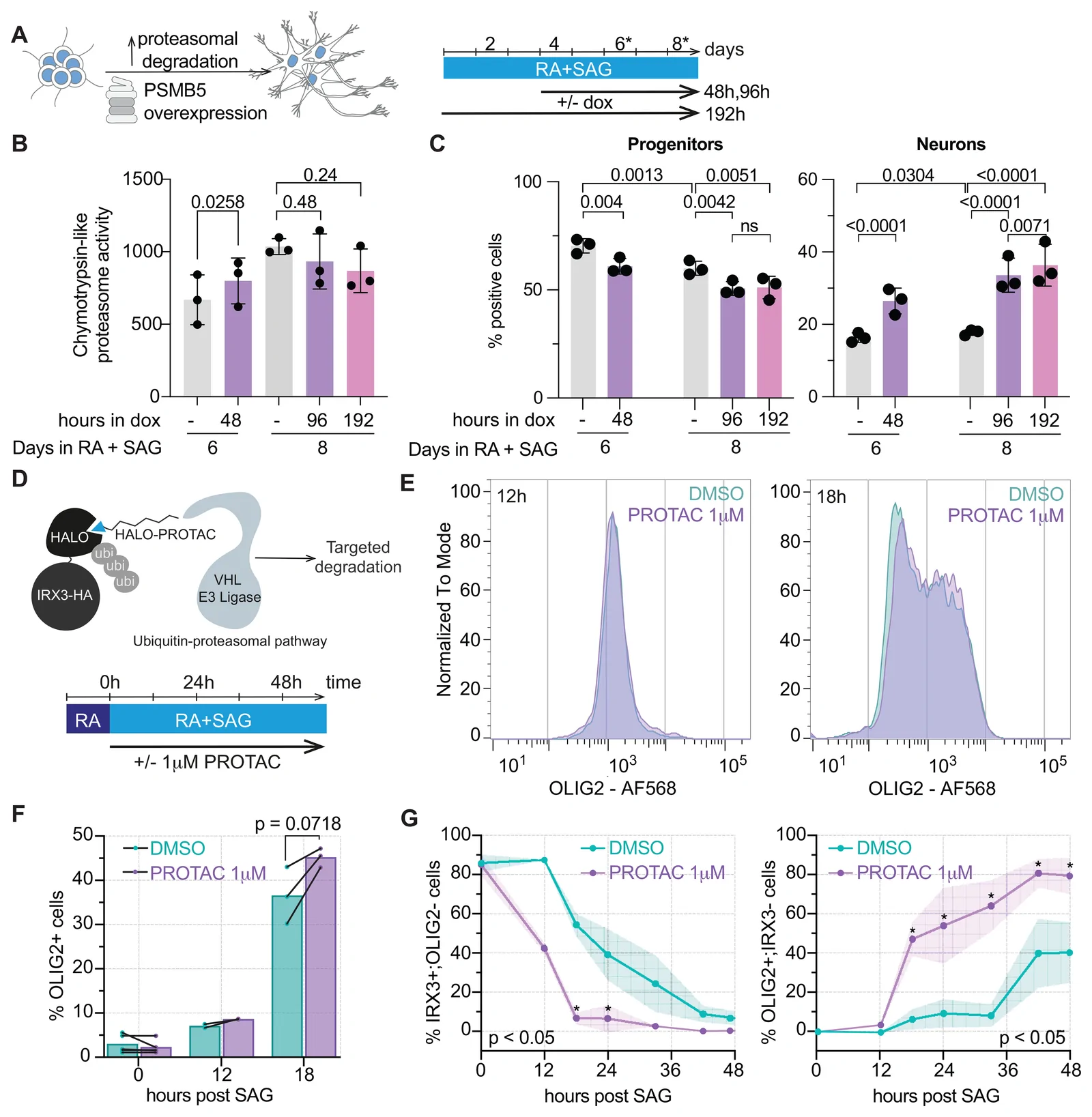
Effects of increased proteasome-mediated degradation rates in human and mouse NPs. (a) Schematic of PSMB5 overexpression in human neural progenitors (hNPs). Doxycycline induced PSMB5 overexpression increased proteasomal activity, when administered from day 0-8 (192 h) or day 4-8 of differentiation. Samples were collected at day 6 (48 h) and day 8 (96 h, 192 h). (b) Proteasomal activity measured as the slope of fluorescence increase from chymotrypsin substrate digestion in human NPs treated with or without doxycycline for the indicated duration at the indicated days of differentiation (n=3). *P* values represent multiple *t*-test comparisons. (c) Quantifications of the proportion of neural progenitors (SOX2^+^), and neurons (TUBB3^+^) treated with doxycycline compared to controls in human NPs for the indicated duration at the indicated days of differentiation. Data represent mean ± standard deviation (n=3). P-values from multiple *t*-test comparisons. (d) Schematics of HaloPROTAC targeted degradation and treatment regimes. HaloPROTAC covalently binds to HALO and induces the 26S proteasomal degradation of IRX3-HALO through the recruitment of the von Hippel-Lindau (VHL) E3 ubiquitin ligase. (e) Representative FACS plots of OLIG2 expression measured in SOX2 expressing mouse NPs in controls (DMSO, turquoise) and PROTAC (+PROTAC, violet) treated samples at 12 h and 18 h post addition. (f) Percentage of total OLIG2^+^ mouse NPs at 0 h, 12 h and 18 h post SAG addition in controls and PROTAC treated samples. Data lines represent biological replicates; p-value is from paired sample t-test (0 h n=5; 12 h n=2; 18 h n=3). (g) Percentage of IRX3^+^;OLIG2^-^ and OLIG2^+^;IRX3^-^ NPs during differentiation under DMSO or PROTAC treatment. Line shows paired mean values (dots), and the shaded areas represent the standard deviation. Two-way ANOVA with Sidak’s post hoc test. Reported P-values from ANOVA results, asterisks indicate post hoc significance (n =5 except 12 h n=2 and 18 h n=3).

Although global proteasome inhibition suggested that an increased stabilisation of the proteome decelerates mouse motor neuron differentiation, the associated proteotoxic stress limited the interpretation. Next, we performed the gain-of-function experiment and tested whether increasing proteasomal activity accelerates human differentiation by overexpressing PSMB5. *Psmb5* encodes the β5 subunit, the rate-limiting catalytic component of the proteasome, responsible for chymotrypsin-like activity and the direct target of the proteasome inhibitor BTZ^42,43^. We introduced an inducible mouse *Psmb5* construct via PiggyBac in hESCs, allowing doxycycline-induced expression of *Psmb5*. We first validated robust upregulation of the exogenous *Psmb5* transcript upon doxycycline treatment (Fig. S5a), accompanied by enhanced proteasome activity (Fig. S5b) in pluripotent cells. Then, cells were differentiated into spinal cord ventral neural progenitors, with doxycycline treatment applied either from day 0 onward or within a defined window (days 4–8) (Fig. 5a). Doxycycline treatment induced significant Psmb5 mRNA upregulation (Fig. S5c), and increased proteasomal activity in hNPs at day 6 after 48 h of treatment (Fig 5b, S5d). Effects at day 8, following 96 h or 192 h of induction, were more variable. This might reflect compensatory feedback, stage-specific differences in proteasomal capacity, or increased cellular heterogeneity. Functionally, PSMB5 overexpression for 48 h (day 6), 96 h (day 8) or 192 h (day 8) increased the proportion of TUBB3^+^ neurons at the expense of SOX2^+^ progenitors (Fig. 5c), indicating that enhanced proteasome activity accelerates the transition from neural progenitors to neurons.

Together, these results provide evidence that decreased proteasomal activity in mouse decelerates motor neuron differentiation and that increased proteasomal activity in humans accelerates the generation of neurons from hNPs.

### Faster IRX3 clearance accelerates OLIG2 expression

Given the widespread effects of global proteasome perturbations, we set out to test whether protein degradation directly affects the rate of the gene regulatory network controlling NP patterning by targeting the degradation rate of a specific transcription factor in the network. Modelling of the network predicts that protein degradation rates can act as quantitative control parameters for developmental tempo^2^. We therefore asked whether acute reduction of protein stability of a single regulatory node could accelerate the temporal progression of NPs, recognising that simultaneous perturbation of multiple network components would be technically challenging. We focused on IRX3 as a representative node. It occupies a key position in spinal cord patterning and is a well-established repressor of OLIG2 and motor neuron progenitor formation. While one study implicates microRNA regulation in this process^35^, the mechanism of IRX3-mediated OLIG2 repression remains unclear^45^, and in vivo overexpression experiments indicate that IRX3 efficiently blocks OLIG2 expression^35^. Thus, IRX3 provides a biologically relevant and experimentally tractable entry point to test whether post-translational control of a key transcriptional regulator via proteasomal degradation can influence the timing of downstream targets, rather than merely their final qualitative outcome. To this end, we generated an Irx3::HA::HALO mouse ESC line (Irx3-HALO) by inserting the HALO tag at the C-terminus of the Irx3 gene. An HA epitope was inserted as a linker between the Irx3 coding region and HaloTag^46^.

We tested Irx3-HALO expression and functionality in spinal cord motor neuron differentiation. We differentiated Irx3-HALO cells, and we exposed cells to RA only on day 3 to promote IRX3 expression. We then performed a pulse-chase experiment with HALO-JF549 ligand (Fig. S5e). Cells were pulsed for 1 h, ligand was then removed, and samples collected 0 h, 4 h and 24 h later. We observed decreasing levels of HALO-JF549 signal during the chase, while the level of IRX3 expression, assayed by HA immunofluorescence and flow cytometry, remained constant across the time course (Fig. S5f,g). This confirmed that the Irx3-HALO line reproduces the expected expression pattern of IRX3 and validates the use of the HA::HALO tag as a multimodal tag to track IRX3 levels and dynamics of turnover.

The HALO tag allowed us to use a small molecule degrader, HaloPROTAC, to induce IRX3 degradation. HaloPROTAC binds covalently to the Halo tagged protein and recruits specific E3 ligases, which promote ubiquitination of the protein and subsequent degradation by the 26S proteasome^46,47^ (Fig. 5d). To test whether HaloPROTAC decreased IRX3 protein stability, IRX3 expression was induced by addition of RA for 24 h as before and cultured thereafter in medium with RA and SAG. HaloPROTAC was added to the Irx3-HALO at the time of RA addition. We collected samples at 24 h, 36 h, and 48 h after addition of HaloPROTAC, as well as in a control Irx3-/- cell line. While IRX3 was detected across timepoints in the untreated control condition (-PROTAC), IRX3 was reduced 24 h after HaloPROTAC addition, and HA intensity was reduced almost to the levels found in the Irx3-/- line 48 h after HaloPROTAC addition (Fig. S5h). These results confirm protein depletion of IRX3 by addition of HaloPROTAC.

We next tested how acute IRX3 degradation impacted the dynamics of OLIG2 expression, a target of IRX3 repression^10,48^. We cultured differentiating Irx3-HALO cells for 24 h in RA to induce *Irx3* in neural progenitors as above. Cells were then switched to media containing RA and SAG either in the presence or absence of HaloPROTAC degrader. This culture regime allowed the maximal expression of IRX3 prior to OLIG2 expression induced by SAG induction. We assayed OLIG2 expression 12-48 h after SAG and HaloPROTAC addition (Fig. 5e,f). In NPs that had not been exposed to HaloPROTAC, SAG-dependent repression of IRX3 and induction of OLIG2 took more than 18 h. By contrast, in cells treated with HaloPROTAC, IRX3 was largely absent by 18 h and an increase in the percentage of OLIG2^+^ cells was apparent (Fig. 5f). Quantifications of the IRX3^-^ ;OLIG2^+^ double population revealed a higher proportion of OLIG2^+^ cells and lower variability in the HaloPROTAC condition compared to control (Fig. 5g). Moreover, fewer cells co-expressed IRX3 and OLIG2 with HaloPROTAC, indicating a more rapid transition from the IRX3^+^;OLIG2^-^ to an IRX3^-^;OLIG2^+^ state (Fig. S5i). Together, these results demonstrate that acute degradation of the transcriptional repressor IRX3 accelerates the appearance of OLIG2 expressing neural progenitor cells, providing evidence that increasing protein degradation can accelerate the tempo of developmental gene regulatory networks.

## Discussion

Here we systematically compare protein dynamics between mouse and human neural progenitors using targeted protein labelling, quantitative mass spectrometry, and genetic perturbations. Together, these approaches provide insight into the mechanisms underlying the differences in developmental tempo between these species. We find a ~1.5 fold-difference in protein stability, consistent with previous assays that measured the overall degradation rate of the proteome by global metabolic labelling^49–53^, and a ~2 fold-difference in the rate of production between mouse and human NPs. Together, these findings suggest that protein translation and degradation rates are different between species and may contribute to differences in developmental timing.

Protein synthesis and degradation shape protein concentration and tissue-specific proteomes in adult cells^54–56^. Yet, their roles in developmental systems, where cells simultaneously change size and growth rate, remain unclear. Differences in protein translation and degradation rates between species result in overall similar protein concentrations in human and mouse NPs, as human cells are larger. Comparable protein densities in the two species have also been reported for presomitic mesoderm cells^5,6^, and align with studies reporting that total protein content generally scales with cell volume^19^, maintaining stable concentrations across different cell sizes^57^ and ploidy^20^. These observations suggest that differentially expressed proteins involved in basal biochemical kinetics underlie the differences in developmental tempo between mouse and human.

Our findings support a role for active protein degradation in controlling developmental tempo. Proteomic analysis suggests enrichment of degradative and metabolic pathways, and protein stability differences persist in post-mitotic neurons, consistent with regulated degradation rather than passive dilution through cell division. Species differences in protein degradation can be partly explained by proteasomal activity: mouse NPs show ~1.5-fold higher chymotrypsin-like proteasomal activity than human NPs, and we also detected ~1.73-fold higher activity in mouse embryonic spinal cord tissue relative to stage-matched human *in vivo* samples. Mouse NPs also contain a higher abundance of major proteasome complexes, consistent with increased activity. In the future, dissecting how mNPs achieve higher proteasome abundance and how distinct regulatory complexes differentially tune proteasome function in mouse and human will be key to understanding how homologous proteins are degraded at different rates.

Human cells show a progressive increase in proteasomal activity during spinal cord differentiation, suggesting stage-dependent proteostatic regulation. This contrasts with previous studies in cortical hNPs and neurons, where proteasomal activity is highest in pluripotent cells and declines during cortical neural differentiation^39,58^. Spinal motor neurons generated through transcription factor–driven cell fate programming have been shown to have less proteasome-dependent protein degradation capacity compared to transcription factor induced cranial motor neurons ^59^. Future work should address whether the differences observed between studies arise from differences in experimental protocols and/or from cell type-specific regulation of proteostatic dynamics.

Perturbing proteasomal activity by proteasome inhibition in mouse NPs or by overexpression of PSMB5 in human NPs altered neuronal differentiation, supporting a causal link between proteasome function and developmental tempo. Moreover, increasing the degradation rate of the NP transcriptional repressor IRX3 accelerated expression of its target gene, OLIG2, consistent with an accelerated transition of intermediate NPs to ventral motor neuron progenitors. This demonstrates that modulating the stability of individual regulatory proteins can have a significant effect on gene expression dynamics and cell fate transitions during development. Moreover, these experiments offer proof-of-concept for the use of the HaloPROTAC system for targeted perturbation of gene regulatory network components that could be broadly applied to dissect gene regulatory mechanisms.

Consistent with our findings, Matsuda *et al*. report a pervasive ~1.5-fold slower rate of protein degradation in presomitic mesoderm^62^, and Swovick *et al*. describe differences in protein turnover across twelve different mammalian species with divergent lifespan^63^. The conservation in different tissues of species-specific differences in protein degradation rates suggests that this may be a fundamental mechanism for evolutionary adaptation of timing in development and homeostasis. Our work extends these observations by identifying differences in both proteasome abundance and the stability of regulatory proteasomal subunits, which may have broad consequences for proteome turnover and developmental tempo.

Autophagy-related pathways did not emerge as central in our global proteomic analysis, potentially because autophagy is primarily regulated through flux rather than steady-state abundance of pathway components^62^. By contrast, Matsuda *et al*. show that 2-deoxyglucose (2DG) shifts mouse proteome stability toward human-like rates, selectively affecting proteasome- and ribosome-associated protein turnover while leaving lysosome-mediated degradation largely unchanged^62^. Together, these findings support a model in which species-specific differences in protein turnover of nuclear and metabolic proteins are primarily driven by the proteasome. However, 2DG can have pleiotropic effects in development beyond energy metabolism^63–66^, including alterations in glycosylation and induction of ER stress^67^, which may also contribute to changes in protein stability. Further work is needed to explore the contribution of autophagy to developmental tempo and species-specific protein turnover differences.

While evolutionary differences have often been attributed to changes in gene regulatory networks through gene duplication or *de novo* acquisition of enhancers^2,68,69^, our results emphasise that post-transcriptional processes, particularly protein turnover, play a crucial role in shaping the temporal dynamics of development. Modulating protein stability could offer a flexible and precise method for globally controlling developmental tempo, without requiring extensive changes to gene regulatory networks or protein sequences. Whether such mechanism accounts for heterochronic changes in traits such as primate cortical expansion or the protracted maturation of cortical neurons remains to be determined^3,69,70^. More broadly, our work highlights the importance of considering stage-specific post-translational and proteostatic regulation in developmental systems and evolutionary divergence, an area that remains poorly understood but likely shaped by strong selective constraints.

In conclusion, our study provides a comparison of protein dynamics between mouse and human NPs, revealing that differences in proteasome-mediated protein degradation are a key driver of species-specific developmental timing. These findings raise questions about how proteostasis is globally regulated across species and its role in evolutionary phenotypic diversity. Future studies exploring how protein turnover is controlled globally and its impact across developmental processes in diverse organisms will be essential for deepening our understanding of the molecular basis of evolutionary change. Understanding these mechanisms will not only contribute to our knowledge of evolutionary and developmental biology but may also inform strategies for manipulating cellular timing in contexts such as stem cell differentiation, regenerative medicine, and homeostasis.

### Limitations of the study

The ubiquitin–proteasome system and the autophagy–lysosome pathway are the two major protein degradation pathways in cells. Although our differential protein abundance analyses are consistent with altered proteasome activity, they remain dependent on normalisation assumptions inherent to quantitative proteomics. Consequently, changes in relative protein abundance should be interpreted with appropriate caution, and future studies using complementary approaches for absolute protein quantification will be important to further validate these findings.

This study focused on proteasome-mediated protein degradation and did not directly examine the contribution of the autophagy-lysosome pathway, which primarily degrades proteins associated with membrane-bound organelles, including mitochondria, lysosomes, Golgi apparatus, and the endoplasmic reticulum ^71^, as well as misfolded proteins. As proteins located in membrane-bound organelles tend to be more stable in human than in mouse NPs, it is possible that species-specific differences in protein degradation also involve the autophagy-lysosome system. Since we did not directly assess autophagic flux or determine the subcellular localisation of key proteins involved in tempo regulation, we cannot fully exclude contributions from additional degradation pathways.

In addition, this work does not rule out the possibility that protein degradation influences developmental patterning in addition to tempo. Our analyses were based on bulk temporal measurements of marker expression over differentiation time and therefore capture population-level dynamics rather than cell state-resolved changes. As a result, we did not explicitly compare degradation or expression dynamics across distinct cellular states within the differentiation trajectory and potential state-specific effects on patterning could not be resolved in this study.

## Resource Availability

### Lead Contact

Further information and requests for resources and reagents should be directed to the lead contact, Teresa Rayon (Teresa.rayon@babraham.ac.uk).

### Materials availability

Mouse and human ESC lines and plasmids generated in this study are available upon request.

## STAR Methods text

### Cell culture and neural progenitor differentiation

The use of human ESCs was carried out in accordance with approvals from the UK Stem Cell Bank Steering Committee (SCSC14-18 to J.B. and SCSC21-46 to T.R.). All cell lines used in this study were confirmed to be mycoplasma negative.

WA09/H9 ESC line (WiCell), and OLIG2::HA::SNAP H9 ESC lines were routinely cultured in Essential 8 medium (Thermo Fisher A1517001) or StemFlex medium (Thermo Fisher A3349401) on 0.5 µg/cm^2^ laminin-coated plates (Thermo Fisher A29249) or 0.5 µg/cm^2^ of Vitronectin (Thermo Fisher A14700), and split using Versene (Gibco 15040066).

Mouse ESCs (HM1, OLIG2::HA::SNAP and IRX3::HA::HALO mouse ESC line) were propagated on mitotically inactivated mouse embryonic fibroblasts (feeders) in DMEM knockout medium supplemented with 1000U/ml ESGRO-LIF (ESG117 Sigma Aldrich), 10% cell-culture validated fetal bovine serum, penicillin/streptomycin, 2 mM L-glutamine (GIBCO) or on 0.1% gelatine in ES medium supplemented with LIF/2i, consisting of 1 μM PD0325901 and 3 μM CHIR99021P (Stem Cell Institute).

Mouse and human NP differentiation was performed following Rayon et al.^4^. For cells grown on ES medium with LIF/2i, 2i was removed at the split prior to differentiation. The γ-secretase inhibitor DBZ was used in experiments to fully differentiate neurons. Cells were treated with 10 ng/µL DBZ (Tocris Biosciences Cat. No. 4489) for 72 h starting at day 2 for mouse and day 5 in human. The proteasome inhibitor bortezomib (R&D, 7282) was used in experiments to stabilize the proteome starting at day 0 after RA and SAG addition at the indicated concentrations. Samples were collected at the indicated timepoints and processed for intracellular flow cytometry (see below). Medium was changed daily.

For IRX3::HA:: HALO mESC differentiation, N2B27 was supplemented with 100 nM RA (R2625 Sigma Aldrich) for neural induction at day 0 and supplemented with RA and 500 nM SAG (566660 Calbiochem) thereafter. To achieve IRX3 depletion, from day 1 onwards, cells were treated with 1 mM HaloPROTAC3 (Promega GA3110) or DMSO as control. Samples were collected after 12 h, 18 h, 24 h, 33 h, 42 h, 48 h of HaloPROTAC or DMSO treatment and processed for intracellular flow cytometry (see below). Medium was changed daily.

### Generation of mouse and human ESC lines by CRISPR

Knock-in cell lines were generated by CRISPR-Cas9–mediated homologous recombination, as in Gouti et al. 2017^73^ and Rayon et al. 2020^4^. Briefly, 2 × 10^6^ cells were electroporated with 2 μg of each plasmid using program A23 of Nucleofector II (Amaxa) and mouse stem cell Nucleofector kit (Lonza DPH-1001) or human stem cell Nucleofector I kit (Lonza VPH-5012). For selection, colonies were first treated with 0.5 mg/ml Puromycin (Sigma P9620) for 2 days followed by 50 μg/ml Geneticin (Thermo Fisher 10131027) selection. Individual colonies were picked and replated to allow expansion and a second round of Geneticin selection. Correct integration of the T2A-HA-SNAPTag transgene was verified using long-range PCRs and Sanger sequencing.

#### Generation of the pNTKV-HA-HaloTag and pNTKV-HA-SnapTag vectors

The coding sequence for the HaloTag was extracted by 2-step PCR amplification with Phusion High-Fidelity DNA Polymerase (NEB, M0530) from the pFC27A HaloTag CMV-neo Flexi vector (Promega, G842A), while, at the same time, the coding sequence for the HA epitope (tatccctatgacgtcccggactatgca) and two stop codons were introduced as part of the 5’ primer used for the amplification process.

For the HA-SnapTag cell line, the coding sequence for the SnapTag was extracted equally by PCR amplification from the pSNAPf commercial vector (NEB, N9183S), while the HA coding sequence and two stop codons were introduced as part of the 5’ primer.

The resulting purified DNA elements were separately cloned, by restriction digestion and inserted with in-Fusion cloning into the pNTKV-T2A-mKate vector^74^.

#### Generation of Olig2::HA::SNAP mouse and human ESC lines by CRISPR

For CRISPR-Cas9–mediated homologous recombination, short guide RNA (sgRNA) sequences (mouse: CGGCCAGCGGGGGTGCGTCC, human: CTGTCGCCAGAACGCGC) were cloned into pSpCas9(BB)-2A-Puro (Addgene pX459 plasmid no. 62988). As donor vector, the HA-SnapTag cassette was inserted at the 3′ end of the Olig2 open reading frame, using 2.83-Kb upstream and 5.04-Kb downstream arms for mouse and 2.78-Kb upstream and 1.98-Kb downstream homology arms for human.

#### Generation of mouse Irx3::HA::HALO ESC line

For CRISPR-Cas9–mediated homologous recombination, short guide RNA (sgRNA) sequence: ATGGTTGAAAAGTTAAGACG was cloned into pSpCas9(BB)-2A-Puro (Addgene pX459 plasmid no. 62988). As donor vector, the HA-HaloTag cassette was inserted at the 3′ end of the Irx3 open reading frame, using 2.32-Kb upstream and 0.34-Kb downstream homology arms.

#### Generation of mouse Irx3 knockout line

For CRISPR-Cas9–mediated non-homologous end joining to generate loss of function by frameshift two short guide RNA (sgRNA) sequences: TCTCTACCCACCCGAACGCC and GGATGTACTGGTATCCGAGC were cloned into pSpCas9(BB)-2A-Puro (Addgene pX459 plasmid no. 62988).

#### Generation of doxycycline inducible PSMB5 hESC line

The CAG-T3G-Psmb5 doxycycline-inducible plasmid construct was generated using the 3G Tet-On system in a backbone containing piggyBac inverted terminal repeat (ITR) sequences. The reverse tetracycline transactivator (rtTA) was expressed under the control of the constitutive CAG promoter. Mouse *Psmb5* (NM_011186.1) cDNA was cloned downstream of the tetracycline response element (TRE), allowing inducible expression upon doxycycline treatment. Construct assembly was performed by VectorBuilder, verified by whole-plasmid next-generation sequencing using Plasmidsaurus and correct sequence integration was verified by RT-qPCR.

Transfection was performed using the Neon Transfection System and Neon Transfection Kit (Invitrogen). H9 hESCs were routinely maintained without antibiotics. One hour before transfection, medium was replaced with E8 supplemented with ROCK inhibitor. For each transfection, 2 µg of CAG-T3G::T2A::Puro-TRE-Psmb5 plasmid and 1 µg of PBase plasmid were mixed in 120 µl Buffer R. Cells were dissociated into single cells using Accutase, washed with PBS, and resuspended in Buffer R containing plasmids. A total of 2 × 10^6^ cells were electroporated at 1200 V with 20 ms pulse width for two pulses using the Neon system. Following electroporation, cells were seeded in E8 medium containing ROCK inhibitor on a Vitronectin coated well. Puromycin selection (0.5 µg/ml) was initiated 6 days after transfection, and colonies were manually picked 4 days later.

### SNAP/HALO labelling for pulse or pulse-chase experiments

For OLIG2:HA::SNAP experiments, mouse and human ESCs were differentiated to NP day 2 and day 6, respectively, as described above. To estimate OLIG2 degradation rates, OLIG2::HA::SNAP cells were incubated for 1 h in RA and SAG medium containing 100 nM SNAP-Cell 647-SiR ligand (NEB S9102S). After two PBS washes, cells were incubated in RA and SAG medium containing 1µM SNAP-Cell Block (NEB S9106S) or 100nM SNAP-Cell TMR-Star ligand (NEB S9105S) for the indicated chase periods. To estimate OLIG2 production rates, cells were incubated for 1 h in RA and SAG medium containing SNAP-Cell Block ligand. After two PBS washes, cells were incubated in RA and SAG medium containing SNAP 647-SiR ligand for the indicated chase periods.

For IRX3::HA::SNAP mouse pulse-chase experiments, cells were labelled for 1 h in medium supplemented with 100nM Janelia Fluor HaloTag Ligand (549, Promega GA111) at day 0 on RA as described above. After two PBS washes, cells were incubated in RA and SAG medium for the indicated chase periods.

After SNAP/HALO ligand treatments, cells were washed once or twice with PBS for degradation or production measurements, respectively, and processed for intracellular flow cytometry or imaging. At least 3 biological replicates per species per timepoint from independent experiments were used. For OLIG2 degradation rates, geometrical mean was measured on OLIG2-positive and negative populations. Half-life estimation was based on the non-linear model fitting in GraphPad Prism version 10.0.0 for MacOS using values normalised by samples not treated with SNAP-cell 647-SiR ligand. Error intervals reported correspond with 95% confidence intervals. Images were analysed in FIJI.

### Intracellular Flow Cytometry

Cells were dissociated with 0.5 ml accutase (GIBCO) and fixed in 4% paraformaldehyde in PBS for 5min. For stainings, 0.5 - 1×10^6^ cells were used. Cells were incubated overnight with an antibody mix on PBST with 1% BSA at 4°C. The following day, and when HA (Cell Signaling C29F4, 1:1000) or OLIG2 (R&D AF2418, 1:800) primary antibody were used, cells were pelleted and incubated with donkey anti goat/rabbit Alexa Fluor secondary antibodies (1:1000) at room temperature for 1 h. Cells were resuspended in 0.5 mL PBS and filtered for data acquisition on LSR Fortessa or Biorad ZE5 and MacsQuant Analysers. For OLIG2 measurements, 10,000 ~ 50,000 total events were recorded. For other experiments 10,000 events gated on SOX2 were recorded. Analysis was performed using FlowJo software. The following fluorophore-conjugated antibodies were used: SOX2-V450 (BD 561610, 1:100), TUBB3-AF488 (BioLegend 801203, 1:100), TUBB3-AF647 (BioLegend 560394, 1:100), PAX6-647 (BD Biosciences 562249, 1:100).

For Cell Trace Violet (Thermo Fisher C34557), 1×10^6^ cells were incubated at the stated concentrations and counterstained with LIVE/DEAD nearIR marker (Thermo Fisher L34975) following manufacturer’s instructions. Cells were quantified live.

### RNA extraction, cDNA synthesis and qPCR analysis

RNA was extracted from cells using a QIAGEN RNeasy kit, following the manufacturer’s instructions. Extracts were digested with DNase I to eliminate genomic DNA. First strand cDNA synthesis was performed using Superscript III (Invitrogen) using random hexamers and was amplified using PowerUp SYBR green (Applied Biosystems). qPCR was performed using the QuantStudio 5 Real-Time PCR System. For mouse samples, expression values for each gene were normalised against b-actin, using the delta-delta CT method. Mouse and human qPCR primers used are listed in Rayon et al 2020. New primers include PSMB5 transgene expression: rBGpA_F: GGCCAATGCCCTGGCTCACAAAT; rBGpA_R: CCAGAAGTCAGATGCTCAAGGGGCT. Data were processed, normalised and plotted using Prism GraphPad.

### Immunofluorescence, microscopy and quantitative image analysis

Cells that were prepared for confocal imaging were grown on 1% Matrigel (Corning, Cat # CLS354230) μ-Slide ibiTreat 4 well plates (Cat # 80426, and immunofluorescence on mouse cells was performed as described previously^4^. Primary antibodies were diluted as follows: rabbit anti-HA (Cell Signaling C29F4, 1:1000), Rat anti-Ki-67 (ThermoFisher 14-5698-82, 1:1000), rabbit anti- cleavedCaspase-3 (Cell Signalling Technology #9661, 1:400), goat anti-Sox2 (R&D systems AF2018, 1:500), rabbit anti-HA (Cell Signaling C29F4, 1:1000). Alexa secondary antibodies (Life Technologies) used throughout this study were diluted 1:1000, and nuclei were counterstained with DAPI.

Cells were imaged using a Leica SP8 confocal microscope equipped with a 20× NA 0.75 dry objective or a Leica Stellaris 8 (BI). For each experiment, all imaging settings (laser power, detector gain, and offset) were kept constant across conditions to ensure comparability between samples. Pixel intensities were adjusted uniformly across entire images in Fiji, using identical adjustment parameters for all images within a given experiment. Immunofluorescence was performed on a minimum of three biological replicates from independent experiments at a 20x magnification. For quantification, DAPI staining was used first to segment the nuclei from images in CellProfiler^75^. After nuclei identification from the DAPI images, the segmented nuclei were used to quantify Ki-67 intensities based on integrated intensity measurements. The objects having greater than or equal to 100 pixel area were gated to remove debris from nuclei. From these gated population, objects having more than 10 Integrated intensity on DAPI channel were selected. Segmented nuclei having more than 10 integrated intensity in Ki-67 channel were considered Ki-67^+^ cells. The cell numbers of Ki-67^+^ cells were plotted and analysed using one way ANOVA in Graphpad prism. For cl-Caspase 3, the integrated intensities and object numbers were plotted from raw values without gating for the size.

### Global protein production measurements

#### AHA uptake experiment

Experiments were performed on differentiated neural progenitors (mouse day 2 and human day 6), as described above. The experiments were performed in the presence of methionine as we reasoned that methionine deprivation might trigger a reduction in protein production and impact protein translation. Spent medium was replaced by 1 mM AHA in differentiation medium supplemented with RA and SAG at 0 h timepoint. Samples were collected at the indicated timepoints for intracellular staining with Click It Cell Reaction buffer kit (Invitrogen C10269) and Alkyne Alexa Fluor 488 (Invitrogen A10267) followed by SOX2-V450.

#### Puromycin uptake experiment

We tested a range of puromycin concentrations and timepoints to identify conditions that do not trigger integrated stress response to the neural progenitors. 0.1 mg/ml puromycin for up to 2 h gave sufficient staining intensity without inducing a cell stress response. Puromycin was applied to the differentiating NPs in differentiation medium supplemented with RA and SAG at 0 h timepoint. Samples were collected at the indicated timepoints for intracellular staining with anti-puromycin Alexa Fluor 488 (EDM Millipore MABE343-AF488) and SOX2-V450 conjugated antibodies.

### Coulter counter–based cell counting and volume analysis

Cells were dissociated with accutase (GIBCO), fixed in 4% paraformaldehyde in PBS for 5min, then stored in PBS. For volume measurement 2×10^6^ cells in PBS were diluted with 10 ml Beckman Coulter ISOTON. Cell number and volume were then measured using a Z2 Coulter counter with an aperture of 100 μm and according to the manufacturer’s instructions. Instrument settings were kept constant between experiments. Data were trimmed using an R script designed to exclude debris and non-viable particles.

### Global proteomics

#### Sample prep

Cells were treated with accutase to detach from cell culture plate, washed with PBS, counted and centrifuged. Cell pellets were frozen in an ethanol/dry ice bath and stored in -80oC until ready to process. Protein extracts were prepared with lysis buffer containing: 50mM Tris pH 8, 1% SDS, complete protease inhibitors (one tablet per 3.5 ml buffer, ROCHE 05056489001) and benzonase (250 U/ml, Pierce 88701). Protein concentration was determined using the BCA assay according to manufacturer’s instructions (Pierce 23227). To prepare peptides 100 µg of proteins per sample were acetone precipitated, washed twice with 80% acetone and solubilised in 100 mM Hepes pH 8 at 1 mg/ml protein concentration. To reduce disulfide bonds and alkylate free cysteine residues samples were treated with 10 mM DTT for 30 min followed by 20 mM Iodoacetamide for 30 min, followed again by 10 mM DTT for 30 min. Proteins were digested with LysC (1 µg LysC per 100 µg of proteins) overnight (O/N) followed by Trypsin (1.33 µg Trypsin per 100 µg of proteins) O/N. Samples were stored at -80°C until ready to proceed with mass spec.

#### Data Independent Acquisition (DIA) analysis on timsTOF Pro2

Peptides were analysed using an Evosep One LC system (EvoSep) coupled to a timsTOF Pro 2 mass spectrometer (Bruker Daltonik GmbH) using a commercial 150-mm analytical column (EV1113 ENDURANCE COLUMN, Evosep Biosystems) and an integrated Captive Spray Emitter (IonOpticks). Buffer A was 0.1% formic acid in water, Buffer B was 0.1% formic acid in acetonitrile. Data was collected using diaPASEF with 1 MS frame and 9 diaPASEF frames per cycle with an accumulation and ramp time of 100 ms, for a total cycle time of 1.07 seconds. The diaPASEF frames were separated into 3 ion mobility windows, in total covering the 400 – 1000 m/z mass range with 25 m/z-wide windows between an ion mobility range of 0.64–1.4 Vs/cm^2^. The collision energy was ramped linearly over the ion mobility range, with 20 eV applied at 0.6 Vs/cm^2^ to 59 eV at 1.6 Vs/cm^2^.

#### DIA Data Processing and Analysis

Data were analysed using DIA-NN (version 1.8) with all settings as default except we omitted normalisation and MBR (match between runs) was used. Specifically, a maximum of one tryptic missed cleavage was allowed, with fixed modifications of N-term methionine excision and carbamidomethylation of cysteine residues. No variable modifications were selected. A *Homo sapiens and a Mus musculus* UniProt database was used for the analysis of the relevant samples. A default threshold of 1% false discovery rate was used at both the peptide and protein level. At least two unique peptides were required to identify a protein. The library-free mode of DIA-NN was used to generate precursor and fragment ions *in silico* from the UniProt database. Additionally, the program generates a library of decoy precursors (negative controls). Retention time alignment was performed using endogenous and iRT peptides, and peak scores were calculated by comparison of peak properties between observed and reference spectra.

#### Data Analysis

The data were analysed in three samples (biological replicates) derived from three bulked samples of mouse or human NPs. Each sample was run three times on the mass-spec (three technical replicates). Protein copy numbers per cell were estimated using the proteomic ruler method^21^, and 1:1 homologs between mouse and human were retained for downstream analysis. The table with homologs was extracted from ensemble using BioMart^76^ and then manually curated to include missing gene names (Table S6). The proteins that were identified in at least 2 samples in each group (human or mouse) were used for downstream analysis. To reduce potential technical biases between mouse and human samples, we performed a global correction of protein abundances by dividing them to the size factors of the samples (total copy number of each sample per the mean of all samples). The average copy number between the non-zero technical replicates were used for differential expression, with zeros being replaced by medians intensities in each group (human and mouse). The abundances were transformed to log2 scale and differential expression was performed with R package limma^77^, using empirical Bayes method for 2 group comparison with standard t-test of the eb.fit function output^78^. The differentially expressed proteins (DEPs) were chosen based on the criteria of ≥ 2-fold change (FC) and the false discovery rate (FDR) adjusted P values for multiple-hypothesis testing with the Benjamini–Hochberg method FDR ≤ 0.05.

The ssGSEA was run on each of 6 samples using the GO:BP database and fast.ssgsea function in R^32^. The redundancy of the similar pathway terms (having high percent of overlapping genes) was reduced with SimplifyGO function of simplifyEnrichment package in R^33^. The clusters/groups of similar terms were defined by SimplifyGO function, with the default parameters. The FDRs of the top pathways (with highest FDR) from different groups of similar terms were plotted, for the comparison between the mouse and human samples. Data can be found in Table S4.

The pathway enrichment analysis was performed separately on mouse- or human-upregulated DEPs. We used David functional enrichment analysis^25^ with annotations from GO:BP, GO:MF, GO:CC, KEGG and Reactome databases. The background was represented by orthologous human or mouse proteins identified in mouse or human samples, respectively (Table S1). The DAVID functional annotation clustering was used to group similar and redundant terms and the top representative terms of each cluster were combined for plotting.

### Dynamic SILAC

#### Cell Culture and Stable Isotope Labelling

Mouse and human ESCs were differentiated as described above until 1^st^ and 4^th^ day of RA and SAG addition, respectively. Then, cells were collected as 0 h timepoint, or continued the differentiation in media containing heavy arginine and lysine until collected after 3 h, 8 h, 24 h and 48 h for mouse NPs, or after 6 h, 24 h, 48 h and 96 h for human NPs in three biological replicates. For the “heavy” condition, DMEM/F12 and Neurobasal were replaced by SILAC DMEM/F12 and SILAC Neural Basal (AthenaES 0423 and 0428) supplemented with methionine and leucine. L-arginine hydrochloride (13C6, 15N4) and L-lysine hydrochloride (13C6, 15N2) (Cambridge Isotope Laboratories CNLM-539-H and CNLM-291-H) were dissolved in PBS and kept at -80°C and were used at the final concentrations of 0.549 mM and 0.648 mM, respectively, based on the concentrations in the original medium. Medium change intervals, seeding densities, coating methods and supplements of inhibitors and growth factors followed the differentiation protocols described above.

#### Sample prep

The cell pellets were lysed in 5% SDS/100mM triethylammonium bicarbonate buffer (Sigma TEAB) by probe sonication and heating. Protein concentration was measured by Pierce BCA Assay (Pierce 23227). 40 µg proteins per sample were taken, reduced by tris(2-carboxyethyl)phosphine hydrochloride solution (Sigma) and alkylated by iodoacetamide (Sigma) then purified by acetone precipitation. Proteins were digested with trypsin (0.8 µg; Sequence modified, Promega) overnight at 30°C in 50 mM ammonium bicarbonate.

#### Data Independent Acquisition (DIA) analysis on Orbitrap Eclipse Tribrid

The LC-MS/MS analysis was on the Orbitrap Eclipse Tribrid mass spectrometer coupled with a U3000 RSLCnano system (both from ThermoFisher Scientific) using DIA mode. 200 ng peptides were first loaded to the trap column (100 µm i.d. x 5 cm) and then separated on an analytical column (75 µm id x 50 cm) using a 120-min gradient from 2 - 30% acetonitrile/0.1% formic acid at a flow rate of 300 nL/min. Both trap column and analytical column were packed in-house with C18 (3 µm, Reprosil C18AQ, Dr. Maisch). MS1 scans were acquired from m/z 390 - 865 using a resolution setting at 120,000, AGC at 400,000 and maximum injection time at 50 msec. MS/MS scans were acquired in the Orbitrap in DIA mode, where the precursor mass range was set at m/z 400 – 855 with isolation windows of 16 m/z and 1 m/z overlap. The scan range was set at m/z 200 – 1800, with a resolution at 30,000, maximum injection time at 54 msec, and relative HCD collision energy at 30.

#### Data Analysis

DIA raw files were analysed using Spectronaut version 18.0 using the spectral library-free directDIA approach, searching against a human or mouse UniProtKB-proteome database (version June 2022). The Pulsar search settings were: Trypsin (specific) with maximum 2-missed cleavage sites; peptide length from 7 to 52; Carbamidomethyl (C) as fixed modification; toggle N-terminal M is true; Oxidation (M) and Acetyl (Protein N-terminus) as variable modifications. FDRs at PSM, peptide and protein level were all set to 0.01. Quantitation was based on peak areas from MS2 data, with channel 1 set for light (label-free), and channel 2 set for heavy peptides (Arg(10) and Lys(8)). All other settings were default.

Data were processed in R (v4.4.1) and RStudio (v2023.06.0+421) and mouse and human datasets were processed in parallel. The fraction of light (channel 1) compared to heavy (channel 2) chain was calculated for each replicate at each timepoint (L/(L+H)). Starting from 8127 and 8509 detected proteins from mouse and human NPs, respectively. Quality check filters were applied to remove proteins that had missing data, <0.85 fraction light at the 0h timepoint, <1000 total expression level over all timepoints, or unstable total expression levels across the timepoints. Out of steady state in expression levels was defined as differences in total expression across the timepoints (Kruskal Wallis p<0.01).

Fraction of light was transformed to a log2 scale, and a linear model was fitted for each protein across the timepoints using all three replicates, with the y-intercept constrained to 0. Those with R2 >0.85 and sigma <0.7 were kept, and the slope of the model was used to calculate protein half-life using the following equation: $t1/2=\frac{\log2(0.5)}{\text{slope}}$

Gene set enrichment analysis on the half-lives was conducted using GSEAPreranked [v4.3.3,]^79,80^ to identify GO:BP, GP:MF, and GO:CC terms enriched in short or long half-lives. Terms were deemed significant if they had a False Discovery Rate (FDR) less than 5% (q<0.05). Revigo^81^ was used to collapse similar terms, and those with a dispensability score <0.5 were retained. Terms with fewer than 25 proteins in the dataset or that had greater than 80% overlapping genes with a term with larger normalised effect size (NES) were removed.

Homologs between mouse and human were extracted from Ensembl v112 using BioMart^76,82^, and those with 1:1 homologs were retained. Gene homologs that matched to multiple Uniprot IDs were removed. Half-life fold difference between mouse and human was calculated (human/mouse) to visualise differences in half-life between the species (Table S11).

To group the proteins by GO terms, at least one of the homologous proteins annotated with descendant terms of following were counted: CC: cytoplasm (GO:0005737), nucleus (GO:0005634), membrane (GO:0016020) and extracellular region (GO:0005576); MF: catalytic activity (GO:0003824), structural molecule activity (GO:0005198), transporter activity (GO:0005215), binding (GO:0005488), molecular adaptor activity (GO:0060090), molecular function regulator activity (GO:0098772), transcription regulator activity (GO:0140110). ATP-dependent activity (GO:0140657); For MF, only the terms with more than 100 proteins were shown, and terms with complete overlap were excluded (Table S12).

### Proteome stability measurements in neurons

AHA pulse and chase experiments were performed as in Rayon et. al 2020^4^. Cells were starved by replacing complete differentiation medium with methionine-free medium for 30 min. Next, 100mM AHA was added to the methionine-free medium for 1 h. To measure protein stability, AHA pulse was removed by washing the cells once with PBS and growing the cells on complete differentiation medium for the course of the experiment. On the indicated days of differentiation and at specific timepoints after AHA removal, cells were processed for intracellular flow cytometry.

AHA-incorporated proteins were labelled using Click-iT™ Cell Reaction Buffer Kit (Thermo Fisher Scientific C10269) on a volume of 150 µl per sample reaction. Estimations of global proteome stability were calculated as in Rayon et al. 2020^4^. Briefly, estimations of proteome stability were obtained by normalizing each individual replicate using an initial exponential fit y(t) = B + C · exp(kt) to determine the baseline B and initial fluorescence intensity C, allowing a comparison of all the replicates together. Exponential fits of the bootstraps of the normalised ensemble were used to construct confidence intervals of the degradation rates. Error intervals reported correspond with 95% confidence intervals. At least 2 biological replicates in technical duplicates per species per timepoint from independent experiments were used.

### Proteasome activity measurements

Mouse and human NPs were washed with PBS once and then scraped in proteasome assay buffer (50 mM Tris-HCl, pH7.5, 5 mM MgCl2, 0.5 mM EDTA, 10% Glycerol, 2 mM ATP and 1 mM dithiothreitol). Then, lysate was homogenized by passing through 27-gauge needles ten times. For the embryo extracts mouse and human embryos were dissected in cold PBS to isolate branchial to lumbar spinal cords which were subsequently homogenised in proteasome assay buffer by passing through 27-gauge needles ten times. Lysate was centrifuged at 10,000 x *g* for 10 min at 4°C and supernatant was aliquoted for storage at -80°C. 20 µg of total proteins per well of 96-well plate or 8 µg of total protein per well of 384 well plate with black walls (Greiner, 655090 or 781090) were diluted in the proteasome assay buffer containing fluorogenic substrates for chymotrypsin-like activity (0.333 mg/mL Z-GGL-AMC, Cambridge Bioscience), caspase-like activity (0.133 mg/mL Z-Leu-Leu-Glu-AMC, Enzo Life Sciences), or trypsin-like activity (0.01666 mg/mL Ac-Arg-Leu-Arg-AMC, Enzo Life Sciences). Fluorescence was measured by PHERA star or Ensight plate reader (360 nm excitation, 430 nm emission, BMG Labtech or Perkin Elmer) every 2 min for 60 cycles at 37°C. Kinetic slopes were measured for the stable 60 min. Protein amount was estimated by BCA assay (Pierce 23227) as in Vilchez *et al*. 2012^39^.

All animal procedures were carried out in accordance with the Animal (Scientific Procedures) Act 1986 under the Home Office project license PP8527846. Human embryonic material (4-6 weeks of gestation) was obtained from the MRC/Wellcome-Trust (grant #006237/1) funded Human Developmental Biology Resource (HDBR57, http://www.hdbr.org) with appropriate maternal written consent and approval from the London Fulham Research Ethics Committee (23/LO/0312) and the Newcastle and North Tyneside NHS Health Authority Joint Ethics Committee (23/NE/0135). HDBR is regulated by the UK Human Tissue Authority (HTA; www.hta.gov.uk) and operates in accordance with the relevant HTA Codes of Practice. This work was part of project no. 200804 registered with the HDBR.

### Native gel western blotting and quantification

Cells were processed as described for the proteasome activity assay. Samples containing 9 µg total protein were mixed with Native Sample Buffer (R&D Systems, 1610738) and separated on 4–15% Mini-PROTEAN TGX precast gels (Bio-Rad, 4561084) under non-denaturing conditions. Electrophoresis was performed for 60 min at 150 V in 1× Tris/Glycine buffer (Bio-Rad, 1610734) supplemented with 0.1 mM EDTA, 5 mM MgCl_2_, 0.5 mM DTT, and 0.5 mM ATP under cooled conditions. Precision Plus Protein Ladder (Bio-Rad, 161-0374) and purified human 20S proteasome (R&D Systems, E-360) were included as references.

Proteins were transferred to 0.45 µm nitrocellulose membranes (GE Healthcare) for 70 min at 100 V in Tris/Glycine transfer buffer containing methanol under cooled conditions. Membranes were blocked with Intercept Blocking Buffer (LI-COR), incubated overnight at 4°C with anti-PSMB6 antibody (Thermo Fisher, PA1-978, 1:1,000), followed by IRDye 800CW donkey anti-rabbit IgG secondary antibody (LI-COR, 1:10,000) for 1 h at room temperature. Washes were performed with TBS containing 0.1% Tween-20, and signals were detected using an Odyssey CLx imaging system (LI-COR).

Proteasome configurations were quantified in ImageJ using regions corresponding to the 20S and higher-molecular-weight complexes. Band intensities were log-transformed prior to analysis.

## Supplementary Material

Fig. S1

Table S1

Table S2

Table S3

Table S4

Table S5

Table S6

Table S7

Table S8

Table S9

Table S10

Table S11

Table S12

## Data Availability

The mass spectrometry proteomics datasets generated in this study have been deposited in the PRIDE database^72^ and will be made publicly available as of the date of publication under the accession numbers PXD054152 (Global proteomics) and PXD053697 (SILAC proteomics). Scripts to analyse global and SILAC proteomics are both found at https://github.com/POTsnake2/Nakanoh-Stamataki_2024. Any additional information required to reanalyse the data reported in this paper is available from the lead contact.
