# Supplementary material for: Proteasome-dependent protein degradation shapes developmental tempo in mouse and human neural progenitors
Source: Dev Cell. Author manuscript; Available in PMC 2026 Oct 16. (PMC7619575; doi:10.1016/j.devcel.2026.07.014)
Supplement: Supplementary file 1 [file EMS218459-supplement-Fig__S1.pdf]

Figure S1

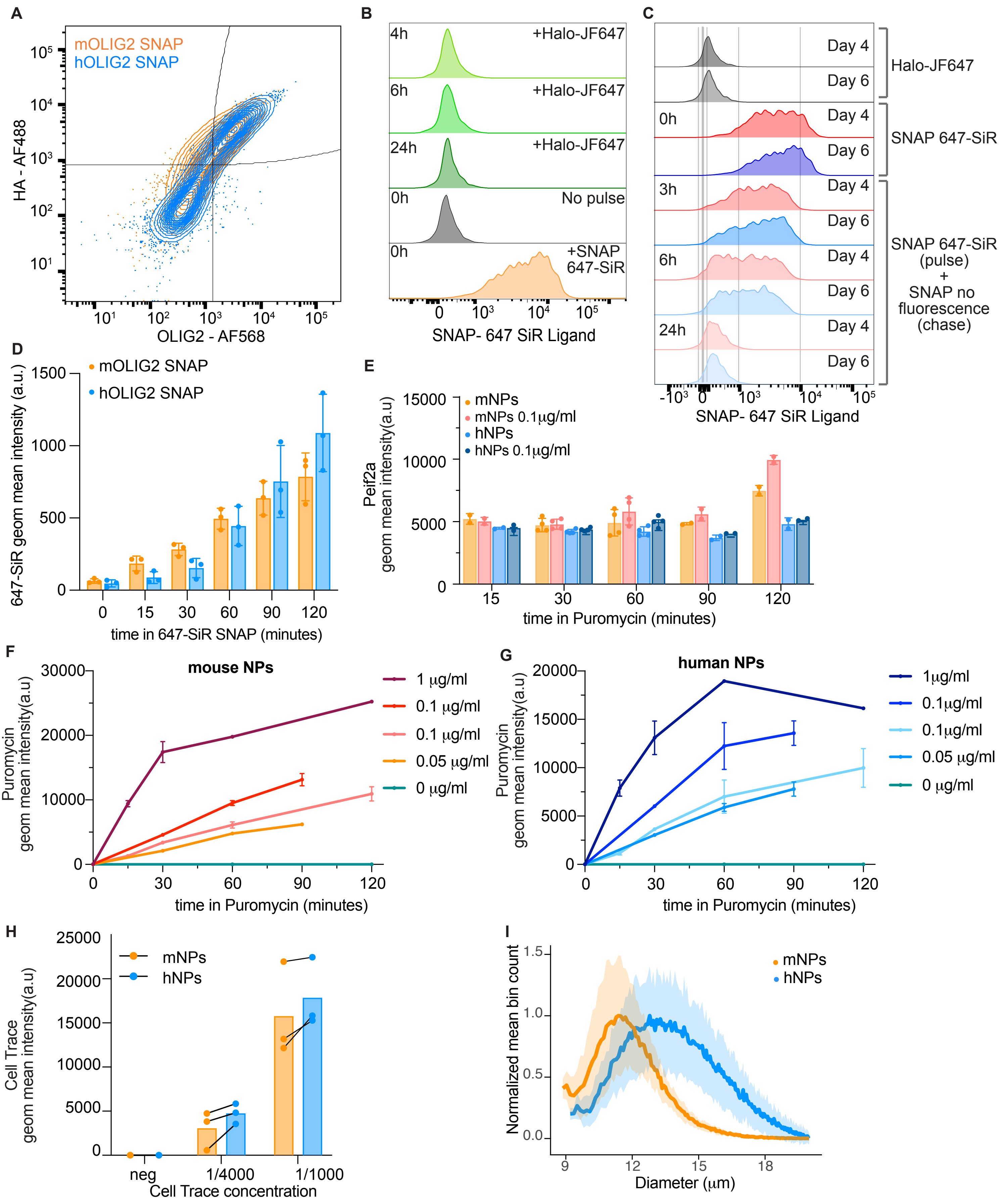

A
mOLIG2 SNAP
hOLIG2 SNAP
HA - AF488
OLIG2 - AF568
B
4h +Halo-JF647
6h +Halo-JF647
24h +Halo-JF647
0h No pulse
0h +SNAP 647-SiR
SNAP- 647 SiR Ligand
C
Day 4
Day 6
Halo-JF647
SNAP 647-SiR
SNAP 647-SiR (pulse) + SNAP no fluorescence (chase)
0h
3h
6h
24h
SNAP- 647 SiR Ligand
D
mOLIG2 SNAP
hOLIG2 SNAP
647-SiR geom mean intensity (a.u.)
time in 647-SiR SNAP (minutes)
E
mNPs
mNPs 0.1μg/ml
hNPs
hNPs 0.1μg/ml
Peif2a geom mean intensity(a.u)
time in Puromycin (minutes)
F
mouse NPs
1 μg/ml
0.1 μg/ml
0.1 μg/ml
0.05 μg/ml
0 μg/ml
Puromycin geom mean intensity(a.u)
time in Puromycin (minutes)
G
human NPs
1μg/ml
0.1μg/ml
0.1μg/ml
0.05 μg/ml
0 μg/ml
Puromycin geom mean intensity(a.u)
time in Puromycin (minutes)
H
mNPs
hNPs
Cell Trace geom mean intensity(a.u)
neg
1/4000
1/1000
Cell Trace concentration
I
mNPs
hNPs
Normalized mean bin count
Diameter (μm)

Figure S1. Assessment of SNAP-Tag suitability to determine protein dynamics
(A) Representative FACS plot showing co-expression of OLIG2 and HA tag in OLIG2-SNAP mouse and human NPs. (B) Representative flow cytometry analysis of 647-SiR ligand incorporation in pulse-chase experiments of human NPs at the indicated days of differentiation with HALO ligands as controls. (C) Representative flow cytometry analysis of 647-SiR ligand incorporation on day 6 (blue) in human NPs pulse-chase experiment. (D) OLIG2-SNAP production measurements in mouse and human NPs. Geometric mean ± standard deviation of SNAP 647-SiR ligand incorporation at the indicated timepoints in mouse and human NPs (n=3). (E) Quantitation of phospho-EIF2A (Peif2a) in mouse and human NPs treated or without puromycin at the indicated timepoints after puromycin incorporation. Data represent mean ± standard deviation (n=2 for 15, 90,120 min and n=4 for 30, 60 min - two experiments with different timepoints). (F) Geometric mean ± standard deviation of fluorescent intensity of conjugated puromycin antibody in mouse NPs treated with the indicated concentrations of puromycin (n=2). (G) Geometric mean ± standard deviation of fluorescent intensity of conjugated puromycin antibody in human NPs treated with the indicated concentrations of puromycin (n=2). (H) Quantification of Cell Trace incorporation of mouse and human NPs as a proxy for protein content (n=3). (I) Coulter counter particle diameter distribution after cell debris removal. Curves normalised measurements per bin. (n=3).

Figure S2

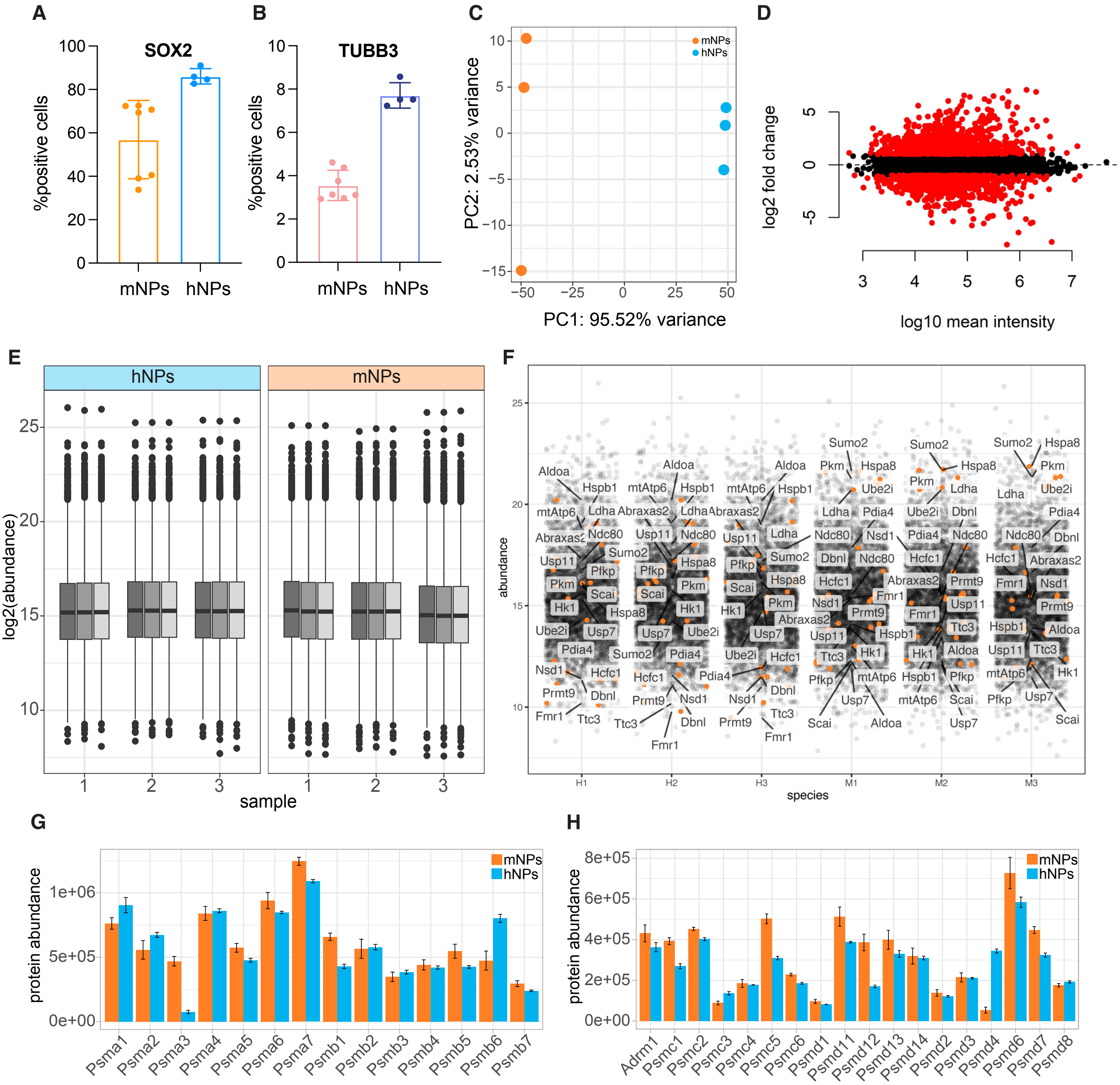

Figure S2. Quality control and differential expression of human and mouse proteins identified by DIA-MS in NPs
(A, B) Percentage of SOX2+ (A) and TUBB3+ (B) cells in mouse and human NP differentiations. Data represent mean ± standard deviation (mouse n=7, human n=4). (C) Principal Component Analysis plot of the mouse and human NPs used for differential protein expression. Each point represents a replicate ran in triplicate for DIA-MS. (D) MA plot displaying the logged intensity ratio (M) versus the mean logged intensities (A) of changes in protein abundances of the mouse and human NPs. The log2 of fold changes in mean human/mouse protein abundance is shown against log10 of the averaged protein abundance. Red dots indicate differentially expressed proteins. (E) Boxplots of the normalised values per technical replicate for the DIA-MS in mouse and human NPs. Each biological replicate was run in triplicate for DIA-MS. (F) Mean abundance of DEPs in mouse and human NPs across the replicates. Proteins mentioned in the main text and shown on the volcano plot are labelled. (G) Mean abundance of core particles of the proteasome in mouse and human NPs as determined by DIA-MS. Data represent mean ± standard deviation (n=3, in triplicates). (H) Mean abundance of regulatory particle subunits of the proteasome in mouse and human NPs as determined by DIA-MS. Data represent mean ± standard deviation (n=3, in triplicates).

Figure S3

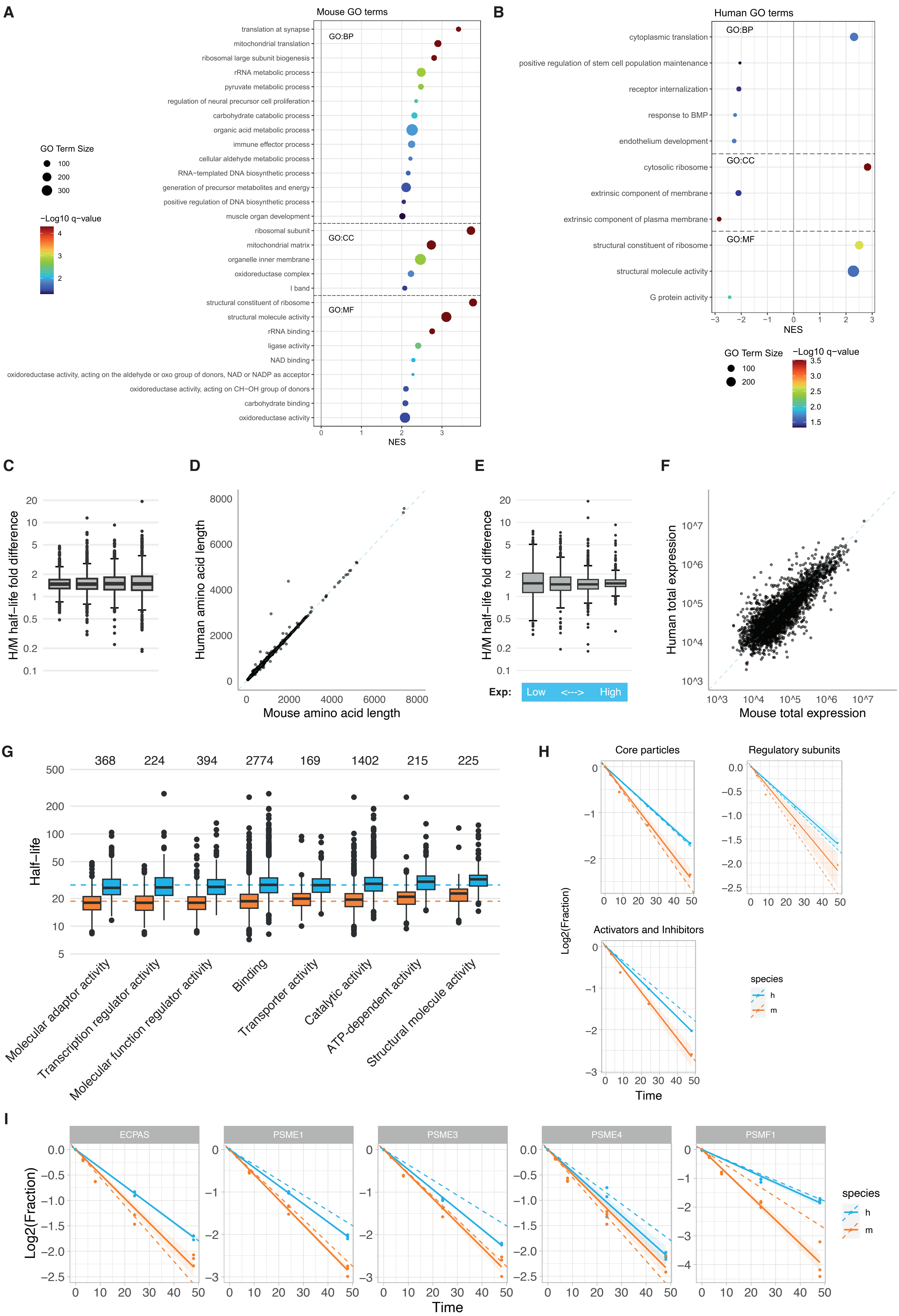

A
Mouse GO terms
GO:BP
translation at synapse
mitochondrial translation
ribosomal large subunit biogenesis
rRNA metabolic process
pyruvate metabolic process
regulation of neural precursor cell proliferation
carbohydrate catabolic process
organic acid metabolic process
immune effector process
cellular aldehyde metabolic process
RNA−templated DNA biosynthetic process
generation of precursor metabolites and energy
positive regulation of DNA biosynthetic process
muscle organ development
GO:CC
ribosomal subunit
mitochondrial matrix
organelle inner membrane
oxidoreductase complex
I band
GO:MF
structural constituent of ribosome
structural molecule activity
rRNA binding
ligase activity
NAD binding
oxidoreductase activity, acting on the aldehyde or oxo group of donors, NAD or NADP as acceptor
oxidoreductase activity, acting on CH−OH group of donors
carbohydrate binding
oxidoreductase activity
0
1
2
3
NES
GO Term Size
100
200
300
−Log10 q−value
4
3
2
B
Human GO terms
GO:BP
cytoplasmic translation
positive regulation of stem cell population maintenance
receptor internalization
response to BMP
endothelium development
GO:CC
cytosolic ribosome
extrinsic component of membrane
extrinsic component of plasma membrane
GO:MF
structural constituent of ribosome
structural molecule activity
G protein activity
−3
−2
−1
0
1
2
3
NES
GO Term Size
100
200
−Log10 q−value
3.5
3.0
2.5
2.0
1.5
C
H/M half−life fold difference
20
10
5
2
1
0.5
0.2
0.1
D
Human amino acid length
Mouse amino acid length
0
2000
4000
6000
8000
E
H/M half−life fold difference
Exp:
Low
<--->
High
F
Human total expression
Mouse total expression
10^3
10^4
10^5
10^6
10^7
G
368
224
394
2774
169
1402
215
225
Half−life
500
200
100
50
20
10
5
Molecular adaptor activity
Transcription regulator activity
Molecular function regulator activity
Binding
Transporter activity
Catalytic activity
ATP-dependent activity
Structural molecule activity
H
Core particles
Regulatory subunits
Activators and Inhibitors
Log2(Fraction)
Time
0
10
20
30
40
50
species
h
m
I
ECPAS
PSME1
PSME3
PSME4
PSMF1
Log2(Fraction)
Time
species
h
m

Figure S3. Additional data analysis of the SILAC dataset
(A, B) Bubble plot showing top enriched GO terms in mouse (A) and human (B) half-lives from GSEA Preranked analysis. Positive normalised enrichment scores (NES) indicate the enrichment in long-lived proteins, and negative NES values indicate enrichment in short-lived proteins. Size of the points depict size of the GO term, colour indicates -log10 p-value. (C) Human to mouse fold difference in half-lives in human homologous proteins partitioned in quartiles according to protein size in human, from small to large. Box-plot: Centre line is the median; box represents upper and lower quartiles; whiskers are1.5x interquartile range from the upper and lower quartiles; dots are outliers. (D) Dot plot showing amino acid sequence length of mouse to human homologues. (E) Quartiles of measured half-lives in human homologous proteins partitioned according to abundance of human proteins, from low to high. Box-plot as in (C). (F) Dot plot showing average total expression levels of mouse and human homologues. (G) Half-lives of mouse (orange) and human (blue) homologous proteins associated with 8 GO molecular function terms. Box-plot as in (C). (H) Median fraction of light over time for proteins related to proteasomal categories in the mouse and human datasets. 14 core proteasomal subunits, 16 regulatory subunits, and 5 proteasomal activators and inhibitors were taken into account. Line and shadowed areas show best linear fit with 95% confidence intervals. Dashed lines represent median protein half-lives. (I) Fraction of light over time for activators and inhibitors of the proteasome in the mouse and human NP SILAC datasets. Line and shadowed areas show best linear fit with 95% confidence intervals. Dashed lines represent median protein half-lives (n=3).

Figure S4

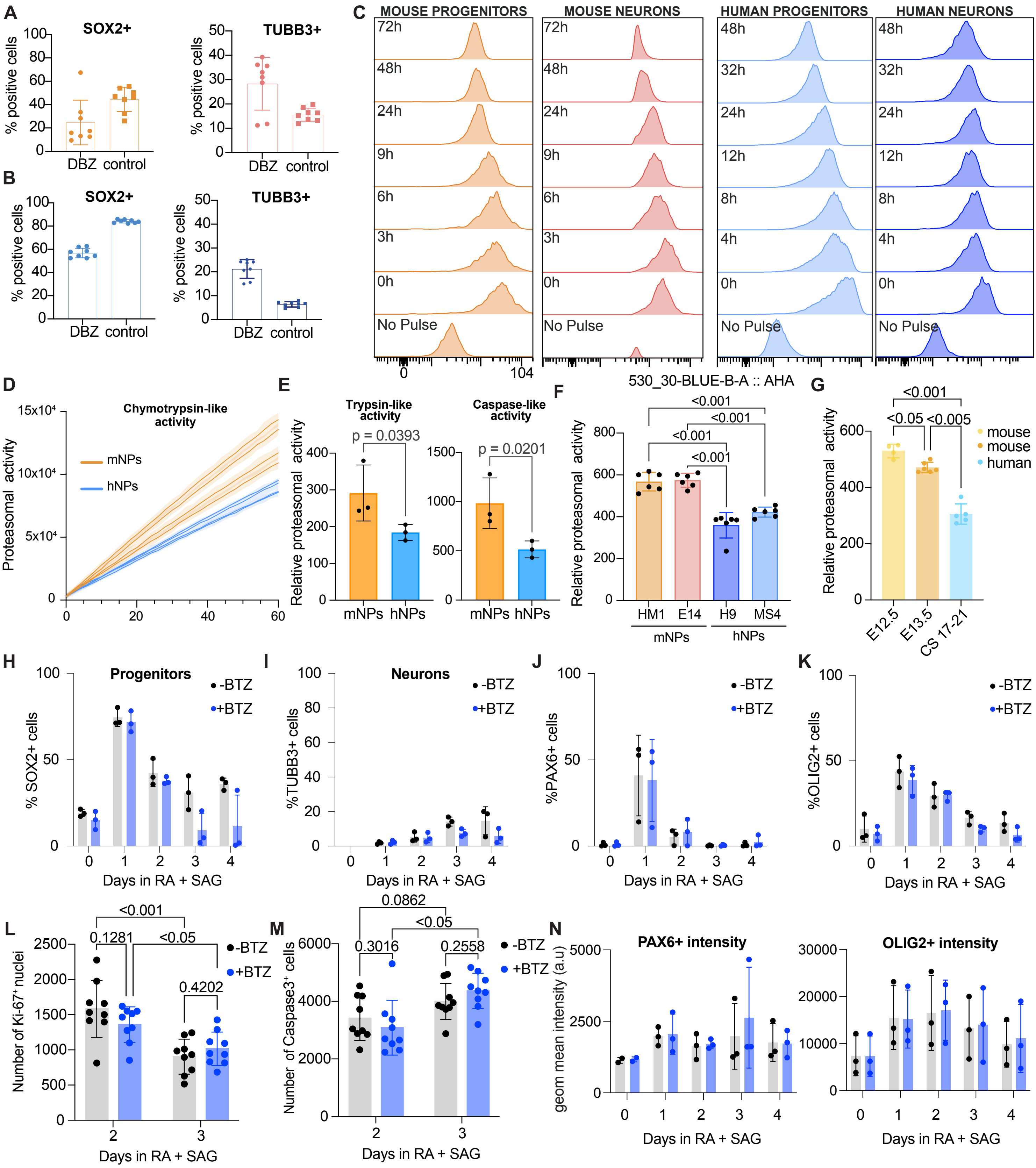

A
SOX2+
TUBB3+
% positive cells
DBZ control
B
SOX2+
TUBB3+
% positive cells
DBZ control
C
MOUSE PROGENITORS
MOUSE NEURONS
HUMAN PROGENITORS
HUMAN NEURONS
No Pulse
530_30-BLUE-B-A :: AHA
D
Chymotrypsin-like activity
mNPs
hNPs
Proteasomal activity
E
Trypsin-like activity
Caspase-like activity
p = 0.0393
p = 0.0201
Relative proteasomal activity
mNPs hNPs
F
HM1 E14 H9 MS4
mNPs
hNPs
Relative proteasomal activity
G
mouse
mouse
human
E12.5
E13.5
CS 17-21
Relative proteasomal activity
H
Progenitors
% SOX2+ cells
-BTZ
+BTZ
Days in RA + SAG
I
Neurons
%TUBB3+ cells
Days in RA + SAG
J
%PAX6+ cells
Days in RA + SAG
K
%OLIG2+ cells
Days in RA + SAG
L
Number of Ki-67+ nuclei
Days in RA + SAG
M
Number of Caspase3+ cells
Days in RA + SAG
N
PAX6+ intensity
OLIG2+ intensity
geom mean intensity (a.u)
Days in RA + SAG

Figure S4. Differences in protein stability between mouse and human neural cells

(A, B) Quantifications of the proportion of neural progenitors (SOX2+) and post-mitotic neurons (TUBB3+) after treatment compared to controls in mouse (A) and human (B) differentiations. Data represent mean ± standard deviation (mouse n=2, human n=2 in duplicates). (C) Representative flow cytometry analysis of AHA incorporation in pulse-chase experiments of mouse and human NPs and neurons at the indicated timepoints. (D) Fluorescence intensity measurements of the chymotrypsin-like enzymatic activity of the proteasome using a fluorescent peptide substrate. A line connects the mean values at the measured timepoints, and the shaded areas represent the standard deviation (n=4). (E) Fluorescence intensity measurements of proteasomal trypsin-like (Ac-Arg-Leu-Arg-AMC) and caspase-like (Z-Leu-Leu-Glu-AMC) activities obtained from fluorogenic peptide substrates. Data are presented as mean ± standard deviation. P-values from paired t-test (n = 3). (F) Proteasomal activity measured as the slope of fluorescence increase from substrate digestion in mouse and human NPs, derived from two mESC and two hESC lines. Data are presented as mean ± standard deviation (n = 6). Statistical analysis was performed using ordinary one-way ANOVA. (G) Proteasomal activity measured as the slope of fluorescence increase from substrate digestion in mouse and human embryonic developing spinal cords. Mouse samples are separated across stages. Data are presented as mean ± standard deviation (E12.5 (n = 4); E13.5 (n = 6); human n = 5. CS17 = 1, CS18 = 1, CS20 =2, CS21=1); Statistical analysis was performed using Brown-Forsythe ANOVA to account for unequal sample sizes. (H-K) Quantifications of the proportion of neural progenitors (SOX2+), post-mitotic neurons (TUBB3+) and early neural progenitor markers (PAX6+, OLIG2+) treated with Bortezomib (5nM BTZ) compared to controls in mouse differentiations. Data represent mean ± standard deviation (n=3). (L) Quantification from confocal images of the number of neural progenitors actively progressing through the cell cycle per image at days 2 and 3 of differentiation following treatment with Bortezomib, measured by Ki-67+ cells. Data represent mean ± standard deviation (n=3), with three independent fields analysed per timepoint and condition. P values represent multiple t-test comparisons, with only control cells at day 3 (- BTZ) showing a significant decrease in proliferation. (M) Quantification from confocal images of the total number of apoptotic neural progenitors per image selected as positive for cleaved caspase-3 at days 2 and 3 of differentiation following treatment with Bortezomib. Data represent mean ± standard deviation (n=3), with three independent fields analyzed per timepoint and condition. P values represent multiple t-test comparisons, with significant differences observed at day 3 between Bortezomib treated (+ BTZ) compared to untreated conditions (- BTZ). (N) Geometric intensity mean for PAX6+ and OLIG2+ neural progenitors treated with Bortezomib compared to controls in mouse differentiations. Data represent mean ± standard deviation (n=3).

Figure S5

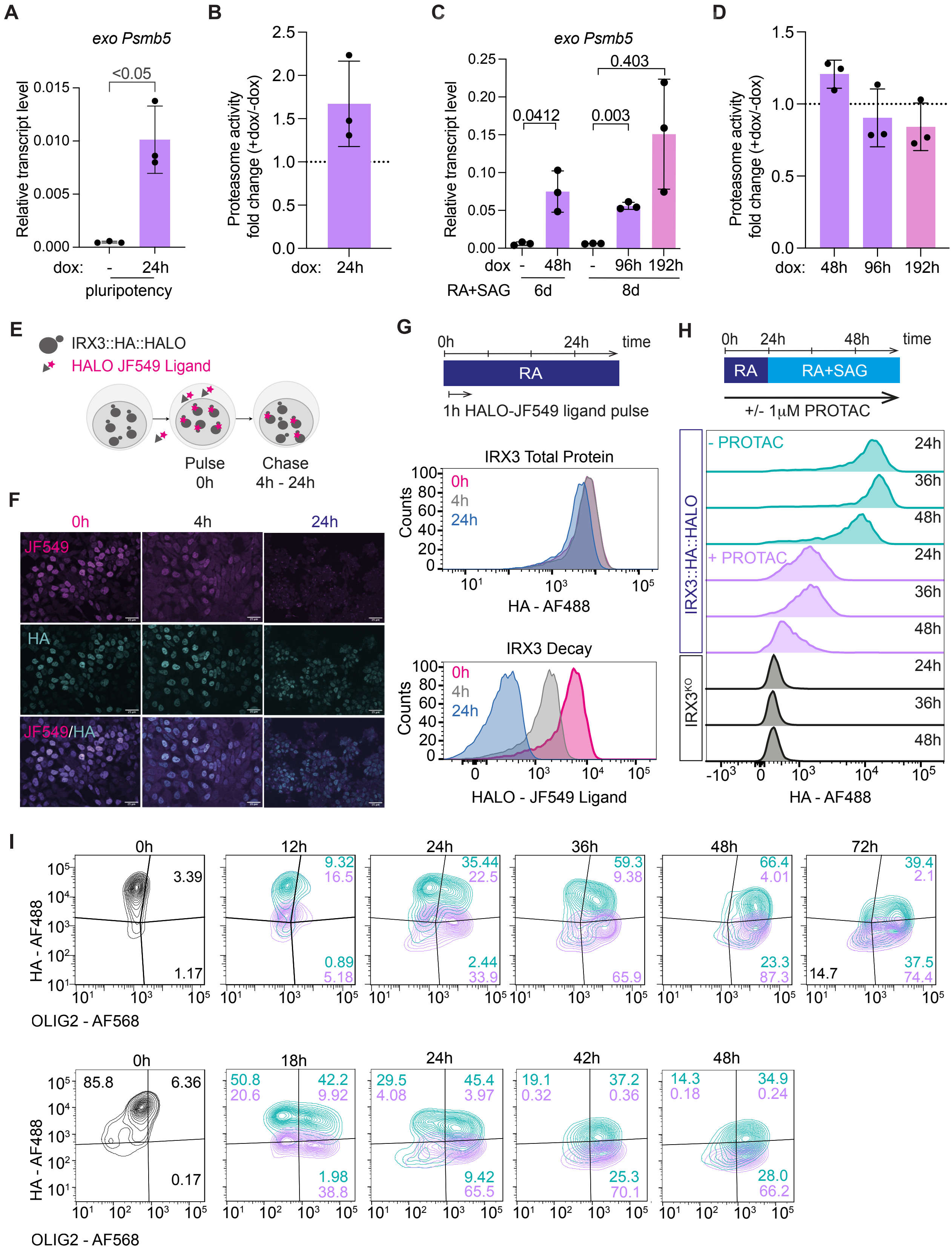
A
exo Psmb5
<0.05
Relative transcript level
0.015
0.010
0.005
0.000
dox: - 24h
pluripotency
B
Proteasome activity fold change (+dox/-dox)
2.5
2.0
1.5
1.0
0.5
0.0
dox: 24h
C
exo Psmb5
0.403
0.0412
0.003
Relative transcript level
0.25
0.20
0.15
0.10
0.05
0.00
dox - 48h - 96h 192h
RA+SAG 6d 8d
D
Proteasome activity fold change (+dox/-dox)
1.5
1.0
0.5
0.0
dox: 48h 96h 192h
E
IRX3::HA::HALO
HALO JF549 Ligand
Pulse 0h
Chase 4h - 24h
F
0h
4h
24h
JF549
HA
JF549/HA
G
0h
24h
time
RA
1h HALO-JF549 ligand pulse
IRX3 Total Protein
Counts
0h
4h
24h
HA - AF488
IRX3 Decay
HALO - JF549 Ligand
H
0h 24h 48h time
RA
RA+SAG
+/- 1µM PROTAC
- PROTAC
+ PROTAC
IRX3::HA::HALO
IRX3KO
24h
36h
48h
HA - AF488
I
0h
12h
24h
36h
48h
72h
3.39
1.17
9.32
16.5
0.89
5.18
35.44
22.5
2.44
33.9
59.3
9.38
65.9
66.4
4.01
23.3
87.3
39.4
2.1
14.7
37.5
74.4
HA - AF488
OLIG2 - AF568
0h
18h
24h
42h
48h
85.8
6.36
0.17
50.8
20.6
42.2
9.92
1.98
38.8
29.5
4.08
45.4
3.97
9.42
65.5
19.1
0.32
37.2
0.36
25.3
70.1
14.3
0.18
34.9
0.24
28.0
66.2
HA - AF488
OLIG2 - AF568

Figure S5. Characterisation of PSMB5-overexpressing hESC and IRX3-HALO mESC line.
(A) RT-qPCR analysis of the exogenous gene (Psmb5) in PSMB5-inducible hESCs comparing untreated and doxycycline-treated conditions (n=3, different clones). (B) Fold change in proteasomal activity in PSMB5-inducible hESCs comparing untreated and doxycycline-treated conditions. Data are presented as mean ± standard deviation (n=3).
(C) RT-qPCR analysis of the exogenous gene (Psmb5) during neural differentiation of PSMB5-inducible hESCs following doxycycline treatment administered from day 0 to day 8 (192 h) or from day 4 to day 8. Data are presented as mean ± standard deviation (n=3). (D) Fold change in proteasomal activity in NPs derived from PSMB5-inducible hESCs comparing untreated and doxycycline-treated conditions. Data are presented as mean ± standard deviation (n=3).
(E) Diagram of pulse-chase treatment for IRX3::HALO ligand. 100 nM of HALO JF549 ligand was added for 1 h. Media was then replaced, and mNPs collected at the indicated timepoints.(F) Confocal images of IRX3 detection and decay from the pulse-chase experiments. IRX3 is detected with an antibody against the HA tag in blue throughout the time course, while the fully labelled IRX3 with the HALO JF549 ligand (magenta) at 0 h shows the decay of IRX3 protein at 4 h and 24 h post-labelling (n=2). Scale bar 25 μm.(G) Schema indicating medium conditions in the pulse-chase labelling experiment and representative flow cytometry histograms of IRX3 expression and decay from the pulse-chase experiments. The histogram on the top corresponds to total IRX3 levels detected with an HA antibody. The lower panel corresponds to the decay of IRX3-JF549 labeled proteins at 4 h and 24 h post-labelling (n=2). (H) Validation of IRX3 depletion by HALO PROTAC as compared to an IRX3KO cell line. Representative histograms of IRX3 expression and depletion over time upon PROTAC addition (n=2).
(I) Representative contour plots of IRX3 depletion in 12 h and 18 h time courses. X-axis represents OLIG2 intensity and y-axis IRX3 intensity. Numbers in each quadrant indicate percentage of NPs quantified in DMSO (turquoise) or PROTAC (violet) conditions.
